# PEEP-Induced Lung Recruitment Maneuver Combined with Prone Position for ARDS: A Single-Center, Prospective, Randomized Clinical Trial

**DOI:** 10.3390/jcm13030853

**Published:** 2024-02-01

**Authors:** Lan Lan, Yuenan Ni, Yubei Zhou, Linxi Fu, Wentao Wu, Ping Li, He Yu, Guopeng Liang, Fengming Luo

**Affiliations:** 1 Department of Respiratory and Critical Care Medicine, West China Hospital, Sichuan University, Chengdu 610064, China; lanlanay@163.com (L.L.); niyuenan0305@scu.edu.cn (Y.N.); zybhh1004@163.com (Y.Z.); fulinxi2022@163.com (L.F.); wuwentaort@163.com (W.W.); liping13258136705@163.com (P.L.); yuhe@wchscu.cn (H.Y.); liangguopeng007@163.com (G.L.); 2 State Key Laboratory of Respiratory Health and Multimorbidity, West China Hospital, Sichuan University, Chengdu 610064, China

**Keywords:** acute respiratory distress syndrome, recruitment maneuver, recruitability, prone position, electric impedance tomography

## Abstract

**Background:** Prone position (PP) and the positive end-expiratory pressure (PEEP)-induced lung recruitment maneuver (LRM) are both efficient in improving oxygenation and prognosis in patients with ARDS. The synergistic effect of PP combined with PEEP-induced LRM in patients with ARDS remains unclear. We aim to explore the effects of PP combined with PEEP-induced LRM on prognosis in patients with moderate to severe ARDS and the predicting role of lung recruitablity. **Methods:** Patients with moderate to severe ARDS were consecutively enrolled. The patients were prospectively assigned to either the intervention (PP with PEEP-induced LRM) or control groups (PP). The clinical outcomes, respiratory mechanics, and electric impedance tomography (EIT) monitoring results for the two groups were compared. Lung recruitablity (recruitment-to-inflation ratio: R/I) was measured during the PEEP-induced LRM procedure and was used for predicting the response to LRM. **Results:** Fifty-eight patients were included in the final analysis, among which 28 patients (48.2%) received PEEP-induced LRM combined with PP. PEEP-induced LRM enhanced the effect of PP by a significant improvement in oxygenation (∆PaO_2_/FiO_2_ 75.8 mmHg vs. 4.75 mmHg, *p* < 0.001) and the compliance of respiratory system (∆C_rs_, 2 mL/cmH_2_O vs. −1 mL/cmH_2_O, *p* = 0.02) among ARDS patients. Based on the EIT measurement, PP combined with PEEP-induced LRM increased the ventilation distribution mainly in the dorsal region (5.0% vs. 2.0%, *p* = 0.015). The R/I ratio was measured in 28 subjects. The higher R/I ratio was related to greater oxygenation improvement after LRM (Pearson’s r = 0.4; *p* = 0.034). **Conclusions:** In patients with moderate to severe ARDS, PEEP-induced LRM combined with PP can improve oxygenation and dorsal ventilation distribution. R/I can be useful to predict responses to LRM.

## 1. Introduction

Acute respiratory distress syndrome (ARDS) is characterized by acute respiratory failure marked by refractory hypoxemia, coupled with bilateral opacities on chest radiographies [1]. The global outbreak of coronavirus disease 2019 (COVID-19) has substantially increased the incidence of ARDS, contributing to an elevated mortality rate [2]. The hallmark pathologies of ARDS involve diffuse pulmonary infiltrates and alveolar flooding, predominantly in gravity-dependent regions [3,4,5]. The functional lung area is reduced due to collapse, regional atelectasis, flooding, or consolidation in ARDS, mainly in the dorsal-dependent lung regions, while normally aerated lungs coexist in the ventral non-dependent regions. Regional lung overdistention and the heterogeneous distribution of stretching forces between ventral aerated and dorsal atelectatic regions increases the risk of ventilator-induced lung injury (VILI) [6,7].

Prone position has been used in critically ill patients since 1970 [8] and is recommended at present for patients with ARDS [9,10,11]. The potential benefit of prone positioning are rooted in mechanisms that include enhancements in ventilation–perfusion matching, reinstating aeration to dorsal lung regions, and facilitating more effective secretion removal processes [12]. It promotes a more uniform lung inflation and an equitable distribution of tidal volume [13]. A series of clinical trials demonstrated a survival advantage associated with prone positioning in ARDS patients [11,14].

The lung recruitment maneuver (LRM), often applied with high positive end-expiratory pressure (PEEP) and airway pressure, is a crucial intervention to improve lung compliance and oxygenation in ARDS patients [15]. It aims to reopen dependent lung atelectasis and improve alveolar recruitment in the lower lobes and/or dorsal lung region [16,17], which can be evaluated by electrical impedance tomography (EIT), a non-invasive and radiation-free clinical imaging technique [18,19].

Studies showed that higher PEEP can be less likely to contribute to regional hyperinflation with proning [20,21]. However, whether prone positioning can act synergistically with the PEEP-induced lung recruitment maneuver (LRM) remains inadequately explored. Limited studies have addressed the potential interactions between prone position and the PEEP-induced LRM, lacking high-quality evidence to substantiate the hypothesis [22,23]. Additionally, it is essential to acknowledge the potential adverse events associated with the LRM, such as hemodynamic deterioration and lung injury. Consequently, predicting the response to a PEEP-induced LRM becomes imperative for discerning that ARDS patients that can derive greater benefits.

In this study, we aim to explore the effect of prone position combined with the PEEP-induced LRM in ARDS patients on the oxygenation and prognosis of ARDS patients, and the predicting role of lung recruitability, measured by the recruitment-to-inflation ratio (R/I).

## 2. Method

### 2.1. Study Design

This was a single-center, prospective clinical trial (ChiCTR2300072905, chictr.org.cn, Registered on 27 June 2023, prospectively registered) conducted in academic ICUs at the West China Hospital of Sichuan University (Chengdu, China), with approval by the relevant institutional research ethics boards. Informed consent was obtained from each patient or their legal substitute decision maker before the onset of any study procedures.

### 2.2. Patients Selection

All mechanically ventilated patients in the ICUs were assessed for the enrollment. The inclusion criteria included (1) an age older than 18 years old; (2) moderate or severe ARDS according to the Berlin definition; and (3) intubation for more than 24 h. The exclusion criteria included (1) contraindications to prone positioning and/or to an EIT assessment and/or to lung recruitment (undrained pneumothorax, severe barotrauma, etc.); (2) hemodynamic instability (>30% increase in vasopressors in the last 6 h or norepinephrine > 0.5 μg/kg/min); (3) PaO_2_/FiO_2_ < 60 mmHg; (4) severe or extremely severe COPD; and (5) clinically suspected elevated intracranial pressure (>18 mmHg).

### 2.3. Data Collection

Throughout the study, all the patients were ventilated with a dedicated ventilator in a volume-controlled mode. The ventilator settings were standardized for all patients during all the study measures: tidal volume (Vt) 6–8 mL/kg of the predicted body weight. The following patients’ characteristics were recorded upon enrollment: demographic data, medical history, and laboratory clinical data. The airway opening pressure was identified by a pressure–volume curve on the ventilator at a low constant flow, as previously described [24].

### 2.4. Intervention

**Figure 1:**
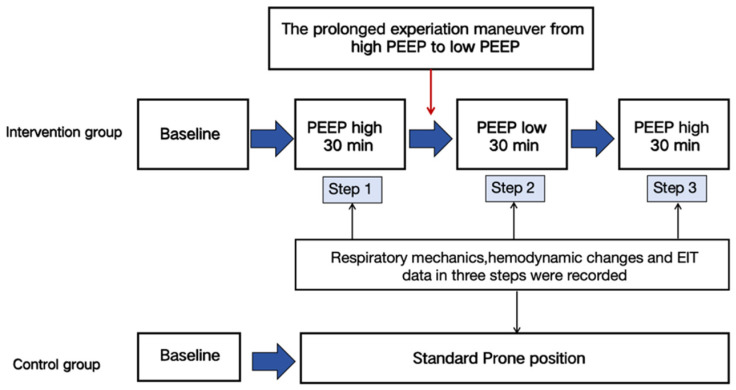
Flowchart of the study.

The patients were prospectively assigned to either the intervention (prone positioning group with a PEEP-induced LRM) or standard control group (prone positioning group) at the clinical decision-making stage within the first prone position session (Supplementary Figures S1 and S2). The random allocation list was generated by a statistician with no clinical involvement in the trial using a computer-generated random number list. After the randomization, patients assigned to the control group continued to receive the prone position for at least 12 h per day according to the guidelines [25]. Each patient in the control group received the prone position at least twice for 3 consecutive days, while patients assigned to the experimental strategy received PEEP-induced LRMs 1 h after the prone position initiation (Figure 1). Then, they continued with the prone position for at least 12 h per day [25]. After the initial PEEP-induced LRM and prone position, the PEEP-induced LRM combined with the prone position was repeated at least 2 times in the intervention group within 3 days. During this process, they were kept under deep sedation and neuromuscular paralysis conditions. Apart from the PEEP-induced LRM, other aspects of care were similar for both groups. The intervention and control group procedures are detailed in the protocol and Supplementary Materials (Supplementary Figure S3 and Supplementary Method S1). To avoid an interference with the immediate effect of the PEEP-induced recruitment, all the respiratory mechanics and other parameters were measured in one hour following the intervention at the clinical PEEP level.

### 2.5. PEEP-Induced Recruitment Maneuver

The PEEP-induced lung recruitment maneuver (LRM) was performed as previously described [24]. Patients were ventilated at two PEEP levels in three steps by order, keeping a 10 cm H_2_O difference between the two PEEP levels (Supplementary Figure S4). Firstly, a high PEEP (PEEP_high_) value was set between 15 to 18 cm H_2_O, lasting for 30 min. After a prolonged deflation maneuver, the PEEP was abruptly decreased to a low PEEP (PEEP_low_) level, which was set at 5 to 8 cm H_2_O, lasting for 30 min. Then, the PEEP_low_ was increased to PEEP_high_ and maintained for 30 min. The flow diagram of the PEEP-induced LRM is shown in Figure 1. Respiratory mechanics and hemodynamic changes were recorded in each step. After these steps, all ventilator settings were returned to those prior to the study. The procedures for the prolonged deflation maneuver are detailed in the Supplementary Method S1. To avoid an interference with the immediate effect of the PEEP-induced LRMs, the PaO_2_/FiO_2_ ratio and respiratory mechanics were measured in one hour following the intervention at the clinical PEEP level.

### 2.6. Electric Impedance Tomography

The EIT data were recorded continuously during the entire procedure. The EIT belt was placed around the chest wall at the fourth or fifth intercostal spaces in both the supine and prone positions and connected to the EIT monitor (PulmoVista 500; Dräger Medical GmbH, Lübeck, Germany). The EIT images were reconstructed based on the tissue impedance variation (delta Z), showing the pixel-level ventilation. The percentage of ventilated pixels in the respective region were named the region of interest (ROI), which were divided into four regions in the horizonal layers. Based on the tidal image of the reference section, the ventilated area was divided into two equally large surfaces: a ventral region (the non-dependent zone) and a dorsal region (the dependent zone) (Supplementary Figure S5). In the control group, the EIT assessment and other respiratory parameters were recorded in the supine position (T0), 3 h after the prone position initiation (T1), and at the end of the prone position (T2). Each recording was performed after the patients were returned to the initial ventilator setting. In the intervention group, the EIT assessment and other respiratory parameters were recorded in the supine position (T0), at the end of the PEEP-induced LRM (T1), and at the end of the prone position (T2). Each recording was performed after the patients were returned to the initial ventilator setting. For each registration point, a stable phase of 30 consecutive breaths (2–3 min) was selected.

### 2.7. Recruitability

The recruitment-to-inflation ratio (R/I ratio) is a new mechanics-based index used to directly quantify the potential for lung recruitment and assess lung recruitability [24]. It is the ratio between the compliance of the recruited lung volume (C_rec_) and respiratory system compliance after a prolonged deflation period, which ranges from 0 to 2.0. As previously described, a threshold of 0.5 was used to characterize high recruiters [24,26]. It was calculated with the expiratory tidal volume measured at the time of releasing a high PEEP to a low PEEP (or airway opening pressure, either of which was higher) in both positions. The compliance of the recruited lung (C_rec_) was defined as the change in the lung volume (∆V_rec_) divided by the effective change in the pressure (PEEP_high_ − PEEP_low_). The recruited volume (V_rec_) was calculated by the difference between the measured ∆EELV and the predicted ∆EELV. The tidal volume (V_T_) released from a high to low PEEP during the prolonged inflation maneuver was calculated, including the exhaled VT at a high PEEP and EELV. The predicted ∆EELV was the product of the compliance of the respiratory system (C_rs_) at a low PEEP and the change in PEEP. The relative formula used is as follows:

(1) $$\frac{R}{I}=\frac{Crec}{Crs\;at\;PEEPlow\;or\;above\;AOP}$$

(2) $$Crec=\frac{Vrec}{PEEPhigh-PEEPlow\;or\;aove\;AOP}$$

(3) ∆Vrec=measured∆EELV−predicted∆EELV

(4) predicted∆EELV=Crs at PEEPlow×(PEEPhigh−PEEPlow)

### 2.8. Statistical Analysis

All statistical analyses were performed by using prism ver. 9.0 (GraphPad Software, San Diego, CA, USA) and SPSS ver. 26 (IBM, Chicago, IL, USA). The Shapiro—Wilk test confirmed a normal distribution. For normally distributed data, the results are expressed as the mean ± standard deviation (SD). When the parameters have non-normal distributions, the data are expressed as the median (interquartile range: IQR). Mann–Whitney U tests (or Fisher’s exact tests for the categorical data) were used to compare the differences between the two groups. The Friedman ANOVA for repeated measures was used to compare the data collected at each step, followed by pairwise comparisons using a Dunn’s post hoc test with the Bonferroni correction. Qualitative data were compared with the chi-squared test or Fisher’s exact test. Kaplan–Meier curves were used to assess the effect of treatment on a 28-day survival rate. Mortality rates were analyzed by the chi-squared test. And, we used Cox proportional hazards to assess the interactions between the treatment effect and the following prespecified subgroups. The correlation between the continuous variables was assessed by the Pearson’s regression coefficient. All the tests were 2-tailed and the differences were considered significant when *p* ≤ 0.05.

## 3. Results

A total of 84 patients with ARDS were assessed for eligibility and 62 patients met the inclusion criteria. Four patients were excluded due to being unable to perform the prone position (2) or due to hemodynamic instability (2). Fifty-eight patients were included in the final analysis, whose main characteristics are summarized in Table 1. Half of the subjects were infected with COVID-19 (48.2%) and the proportion of pulmonary origin cases equaled the extrapulmonary origin (25.9%). The details are listed in the Supplementary Table S1.

### 3.1. Respiratory Mechanics and Gas Change

**Figure 2:**
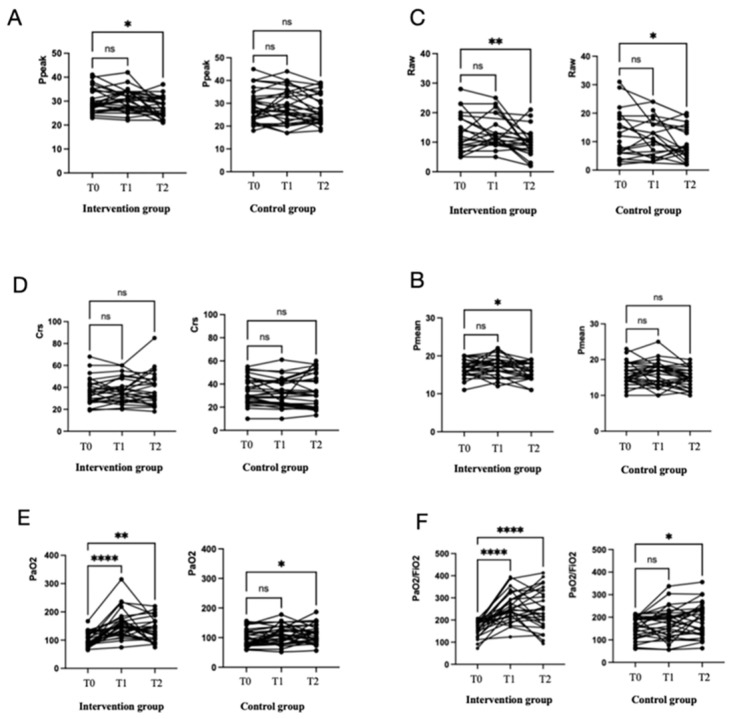
Effects of the prone position combined with the LRM in the intervention group on respiratory mechanics and gas exchanges, compared with the prone position without an LRM in the control group during the first prone position. Effects of the LRM on the peak pressure and mean pressure (**A**,**B**), R_aw_ and respiratory compliance (**C**,**D**), and PaO_2_ and PaO_2_/FiO_2_ (**E**,**F**). * *p* < 0.05, ** *p* < 0.01, **** *p* < 0.0001. ns, not significant.

A total of 28 patients (48.2%) received a PEEP-induced lung recruitment (LRM) combined with the prone position after randomization. In the intervention group, the P_peak_ (28.5 (26.25, 34.75) cmH_2_O vs. 26.5 (24.0, 31.0) cmH_2_O *p* = 0.002) and P_mean_ (17 (15, 18) cmH_2_O vs. 16 (14, 18) cmH_2_O, *p* = 0.019) decreased significantly after the prone position. The PaO_2_/FiO_2_ ratio increased significantly after the prone position in the intervention group (177 (141.6, 194.8) mmHg vs. 238.3 (176.4, 328.7) mmHg, *p* < 0.001) and control group 172.3 (122.5, 195.1) mmHg vs. 193.3 (122.8, 238.0) mmHg, *p* = 0.02) (Table 2) (Figure 2). However, in the control group, P_peak_ and P_mean_ were not significantly modified after the prone position. Respiratory mechanics and oxygenation parameters for three consecutive interventions/days were calculated; the comparisons of changes in the two groups are detailed in Supplementary Materials (Supplementary Tables S2 and S3).

**Figure 3:**
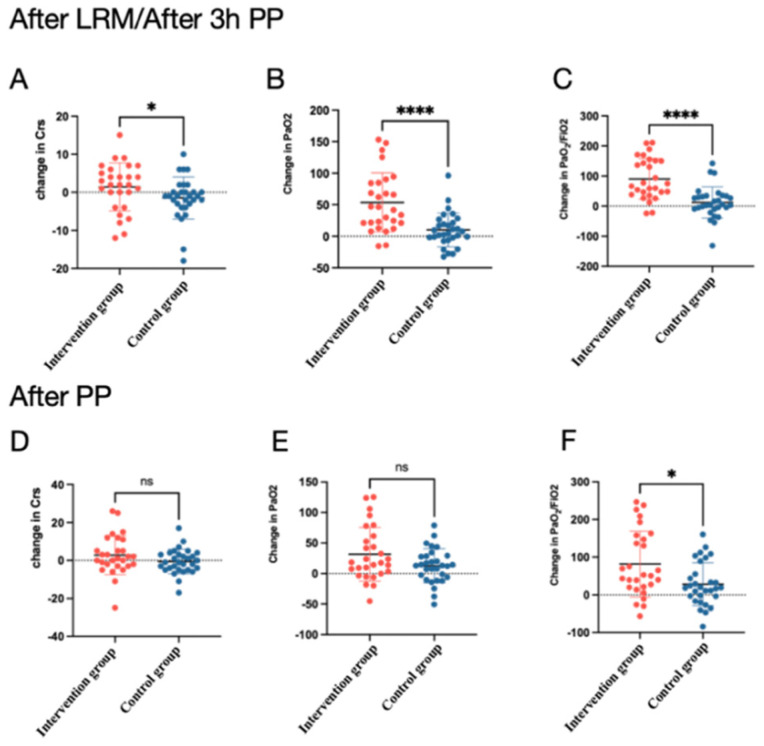
The changes in C_rs_, PaO_2_, and PaO_2_/FiO_2_ in intervention groups after PEEP-induced lung recruitments or the control group after 3 h in the prone position (**A**–**C**); change in Crs, PaO_2_, and PaO_2_/FiO_2_ in intervention group or control group after being in the prone position (**D**–**F**). * *p* < 0.05, **** *p*< 0.0001; ns, not significant.

During the prone position, the PEEP-induced LRM led to a significant change in the compliance of the respiratory system (∆C_rs_) (2 (−3, 5.75) mL/cmH_2_O vs. −1 (−3.25, 0.5) mL/cmH_2_O, *p* = 0.024) and a change in the PaO_2_/FiO_2_ ratio (∆PaO_2_/FiO_2_ ratio 75.8 (36.6, 153.4) mmHg vs. 4.75 (−9.28,4.75) mmHg, *p* < 0.001) (Supplementary Table S4) (Figure 3). After the prone position, the PaO_2_/FiO_2_ ratio remained stable in the intervention group compared with the control group (∆PaO_2_/FiO_2_ 51.65 (20.2, 155.4) mmHg vs. 18.50 (−6.80, 63.75) mmHg, *p* = 0.01, respectively). There was no significant change in other respiratory and hemodynamic parameters. A subgroup analysis of COVID-19- and non-COVID-19-associated ARDS is also presented in the Supplementary Materials (Supplementary Table S5). The PaO_2_/FiO_2_ ratio and PaO_2_ significantly increased after LRMs in both subgroups.

### 3.2. EIT Data

**Figure 4:**
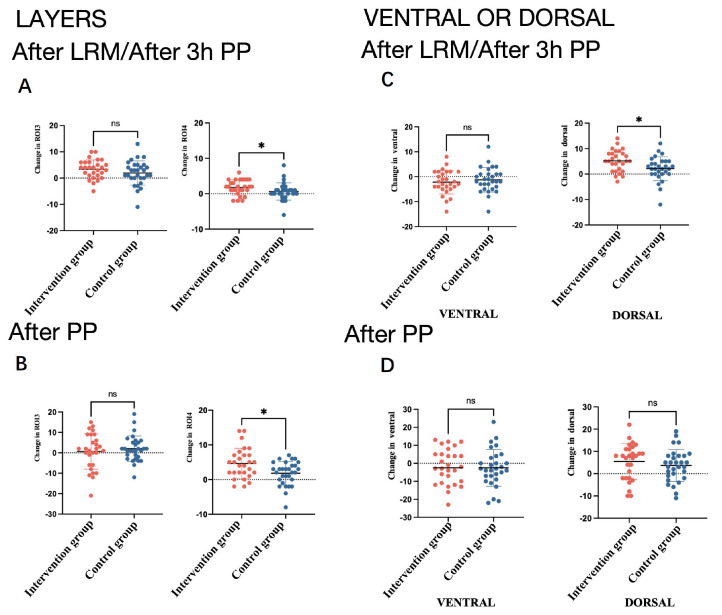
The changes in TV ROI 3 and ROI 4 in the horizonal layers in two groups after the LRM (**A**). The changes in TV ROI 3 and ROI 4 in the horizonal layers in two groups after the prone position (**B**). The changes in the ventral and dorsal regional distributions in two groups after the LRM or after 3 h in the prone position (**C**). The change in the ventral and dorsal regional distributions in the two groups after being in the prone position (**D**). * *p* < 0.05.

The EIT-based measurements of lung ventilation at the indicated time points are detailed in Supplementary Materials (Supplementary Table S6 and Supplementary Figure S6). Compared with the control group, PEEP-induced LRMs led to a greater change in dorsal ROI 4 of the ventilation distribution in the horizonal layers (∆ROI 4 2 (1, 4)% vs. 0.5 (−0.25, 1.25)%, *p* = 0.027) and in the dorsal regional distribution of ventilation (5 (1.25, 8)% vs. 2 (0, 5)%, *p* = 0.015) during the prone position scenario (Supplementary Table S7). The potential of dorsal ROI 4 to perform ventilation distributions in the horizonal layers was evident in the intervention group compared with the control group after employing the prone position (4.5 (2, 7)% vs. 2.5 (−0.25, 5)%, *p* = 0.03) (Figure 4).

### 3.3. Mortality

**Figure 5:**
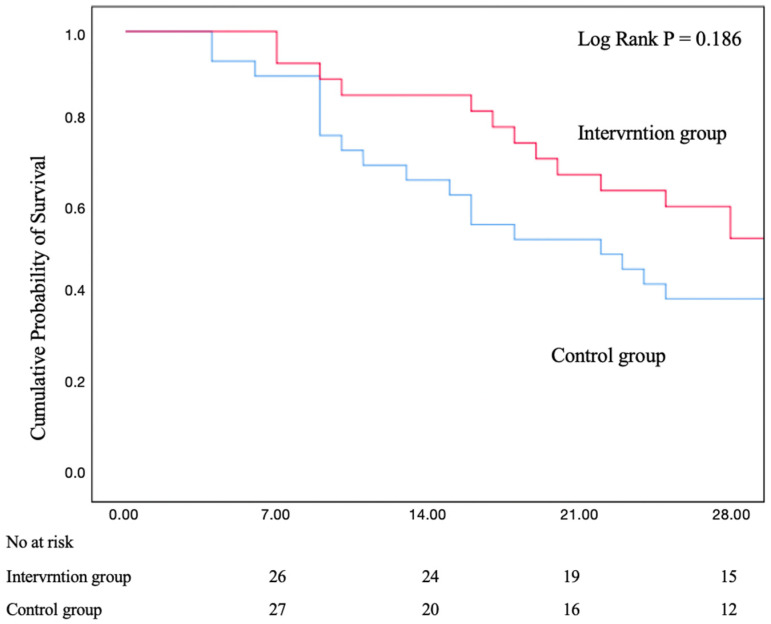
Kaplan–Meier plot measuring 28 days of the probability of survival.

The mortality at day 28 was numerically lower in the prone position combined with the PEEP-induced LRM than in the prone group: 46.4% versus 60.0% (*p* = 0.30) with no statistically significant difference (Table 3 and Figure 5). The lengths of hospital and ICU stays were significantly higher in the prone position combined with LRM group (Table 3). The duration of invasive ventilation and the incidence of adverse effects were similar between the two groups. There was no evidence of the heterogeneity of treatment effects in other subgroups. Treatment effects were also not significantly different according to the type of ARDS (*p* = 0.63 for interactions) (Supplementary Table S8).

### 3.4. R/I Ratio

The R/I ratio was assessed for 28 patients during the PEEP-induced lung recruitment in day 1 (Supplementary Table S9). Among the 28 patients, 16 (57.1%) were considered to be high recruiters according to the threshold of the R/I ratio (0.78 (0.69, 0.95)), and 12 were considered to be low recruiters (0.33 (0.25, 0.44)); the recruitment volume during the PEEP-induced LRMs was significantly higher in the R/I > 0.5 group than the R/I < 0.5 group (608 (525, 721) mL vs. 444 (384, 541) mL, *p* = 0.012) (Supplementary Table S9). The physiologic variables of the two subgroups at the indicated time points are detailed in the Supplementary Materials (Supplementary Table S10 and Supplementary Figure S7). The absolute change in PaO_2_/FiO_2_ was significantly higher in patients with high lung recruitability outcomes than the ones with low lung recruitability outcomes after LRMs (∆PaO_2_/FiO_2_, 140.5 mmHg vs. 47.60 mmHg, *p* = 0.016) (Table 4).

**Figure 6:**
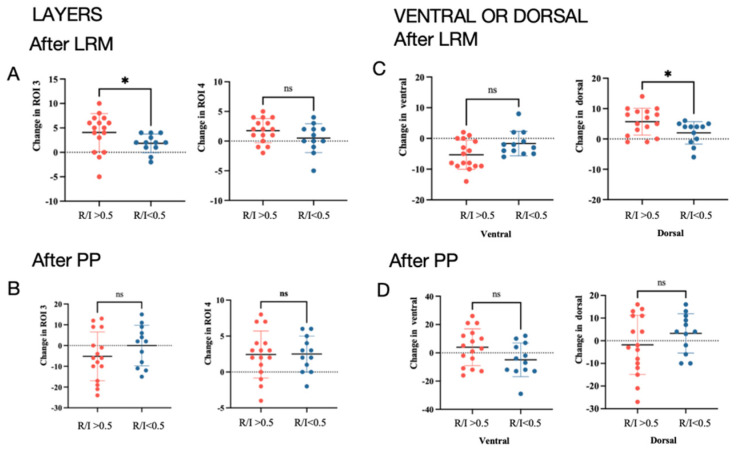
The changes in TV ROI 3 and ROI 4 in the horizonal layers in R/I > 0.5 and R/I < 0.5 groups after LRMs (**A**). The changes in TV ROI 3 and ROI 4 in the horizonal layers in the two groups after being in prone positions (**B**). The changes in ventral and dorsal regional distributions in the two groups after LRMs or 3 h after being in prone positions (**C**). The changes in ventral and dorsal regional distributions in the two groups after being in prone positions (**D**). * *p* < 0.05.

Based on the EIT measurements, the absolute change in the ventilation distribution in the dorsal region was higher in the R/I > 0.5 group than in the R/I < 0.5 group after LRMs (6.0 (1.5, 9.0)% vs. 4.0 (−0.75, 4.75)%, *p* = 0.04) (Table 4) (Figure 6). The images of the regional distribution and tidal impedance variation in the two subgroups are presented in the Supplementary Materials (Supplementary Figures S8–S10). The R/I ratio was positively correlated with the improvement in PaO_2_/FiO_2_ after PEEP-induced LRMs (Pearson’s r = 0.4; *p* < 0.05) (Supplementary Figure S11); the correlation of the R/I ratio and change in the dorsal region distribution (%) were not statistically significant.

## 4. Discussion

Our study explored the effect of PEEP-induced LRMs during prone positions on patients with moderate to severe ARDS. The main findings can be summarized as follows: a PEEP-induced LRM during a prone position could enhance the effect of the prone position by significantly improving the oxygenation; the ventilation distribution mainly occurred in the dorsal region measured by the EIT among ARDS patients; the R/I ratio was positively correlated with the improvement in the oxygenation (PaO_2_/FiO_2)_ induced by the LRM.

In ARDS, lung collapse and regional atelectasis commonly manifest in the dorsal area due to gravity-dependent effects, resulting in decreased respiratory compliance, an increased ventilation/perfusion (V/Q) mismatch, and physiological dead space [2,3]. It contributes to severe gas-exchange impairments and reduced oxygenation. Prone positioning induces regional recruitment in the collapsed dorsal regions and compromises the density distribution of an edematous lung. It can lead to improved oxygenation by improving V/Q matching, increasing the compliance of the respiratory system, decreasing alveolar dead space, and restoring adequate lung gas exchange [4,27,28]. Our present study confirmed that the prone position could improve oxygenation among ARDS patients (172.3 mmHg vs. 193.3 mmHg, *p* = 0.02). Additionally, the PEEP-induced LRM emerges as a potentially beneficial intervention in ARDS, reopening collapsed or poorly aerated alveolar units, reducing intrapulmonary shunts, and increasing pulmonary compliance. PEEP-induced LRM exhibits a synergistic effect combined with the prone position in improving dorsal lung recruitments based on EIT measurements (4.5% vs. 2.5%, *p* = 0.03) and enhancing oxygenation among ARDS patients (75.8 mmHg vs. 4.75 mmHg, *p* < 0.001), compared to the prone position alone. These results can be associated with an increase in well-aerated lung tissue after a PEEP-induced LRM combined with the prone position [27]. A previous study conducted by Marc Gainnie et al. reported that PEEP and prone positioning presented additive effects with a significant increase in PaO_2_/FiO_2_ [29]. Gilles Rival et al. also demonstrated that a high PIP combined with an extended exhalation performed in the prone position could induce progressive alveolar recruitment and a more homogeneous ventilation distribution throughout the lung parenchyma [22]. PEEP-induced LRMs reinforced the recruitment effect of the prone position and promoted a uniform pressure distribution with increases in the PEEP and airway pressure, consistent with the previous literature [22,29,30,31]. On account of the heterogeneity of ARDS types in the studied patients, we performed a subgroup analysis of COVID-19-associated and non-COVID-19-associated ARDS patients. COVID-19-associated ARDS is a special type of ARDS with different heterogeneous phenotypes, including types L and H [32,33]. It has been suggested that type L is not suitable for LRM due to its low lung recruitability and low elastance [34], while type H has the potential for recruitment for its high lung recruitability [33,35]. Our results show that PEEP-induced LRM combined with PP increased the oxygenation and dorsal ventilation distributions among COVID-19-associated ARDS patients. However, we did not classify the COVID-19-associated ARDS phenotypes in the patients included in our study. Recently, Fossali et al. reported that the prone position induced extensive lung recruitment and increased oxygenation among COVID-19-associated ARDS, which was consistent with our results [36]. Further high-quality clinical studies are needed to explore the effect of LRMs combined with PP on different COVID-19-associated ARDS phenotypes.

PEEP during LRMs can also result in hyperinflation and alveolar overdistention, recognized as the key factor of VILI [7,37], especially in patients with the non-recruitable phenotypes [38]. In a supine position, a high PEEP can lead to higher plateau pressures and hyperinflation in already-open tissues. However, in the prone position, the posterior pleural pressure becomes more negative compared with the anterior, mitigating anterior alveolar hyperinflation and reducing the related adverse effects when applying a high PEEP. In our study, the interaction between the prone position and PEEP-induced LRMs could induce a more uniform distribution of ventilation and improved respiratory mechanics, decreasing the airway pressure induced by anterior alveolar hyperinflation, which prevented PEEP-induced VILI. PEEP-induced LRMs combined with the prone position led to a significant increase in respiratory system compliance (C_rs_) compared with the prone position group (*p* = 0.024). The PEEP-induced LRM combined with prone position group also presented a significant decrease in airway pressure after being in the prone position, including Ppeak (from 28.5 cmH_2_O to 26.5 cmH_2_O, *p* = 0.002) and Pmean (from 17 cmH_2_O to 16 cmH_2_O, *p* = 0.019), which is not observed in the prone position group. Above all, our findings indicate that PEEP-induced LRMs applied to prone position group can enhance the recruitment benefit of LRMs but reduce the adverse effects of LRMs.

The LRM is often used in clinical practice but controversies persist regarding its risk/benefit ratio [39]. The mechanisms underlying the inconclusive responses to PEEP-induced LRMs may associated with lung recruitability [35]. Lung recruitability can be assessed by the R/I ratio, which is positively associated with high lung recruitability [24,26]. Previous studies reported that LRMs in poorly recruitable patients had less advanced effects and could even increase lung injuries through excess stress and strain [5,40]. Our findings report a significant correlation between the R/I ratio and improvement in oxygenation by PEEP-induced LRMs (Pearson’s r = 0.4; *p* < 0.05). Responses to PEEP-induced LRMs depend on the R/I ratio, as only high recruiters show a greater change in the dorsal ventilation distribution based on the EIT data (6.0% vs. 4.0%, *p* = 0.04) and a greater improvement in oxygenation (∆PaO_2_/FiO_2_ 140.5 mmHg vs. 47.60 mmHg, *p* = 0.016). The R/I ratio can differentiate the responses to PEEP-induced LRMs in ARDS patients, which can have more benefits for high recruiters. This effect can be explained by the R/I ratio, reflecting the proportion of the volume distributed into the recruited lung and into the baby lung when the PEEP changes during a PEEP-induced LRM, assessing the potential for recruitment [24,41]. The higher the R/I ratio, the greater the volume that aerates into the recruitable lung region rather than the baby lung, which can be used to differentiate patients suitable for LRMs. A previous study proved that a lower R/I ratio was associated with a higher risk of overdistention [26]. In our high recruiter group, the presence of recruitment was not accompanied by a statistically significant increasement in respiratory system compliance, which was consistent with what was previously reported for COVID-19 patients [24,42]. The possible mechanism can be explained as follows. First, the increased volume represented an increase in the compliance of the regional recruited lungs in high recruiters, while the compliance monitored by the ventilator represented the whole respiratory system’s compliance instead of regional lung’s compliance. Thus, it may not depict the regional behavior of lung tissue. Second, baby lung hyperinflation may exist and tidal recruitment at a low PEEP can occur. The compliance of the recruited lung can therefore be lower than that in the regions of pre-existing baby lungs. So, a pre-evaluation of lung recruitability is suggested in order to optimize the use of LRMs in ARDS patients.

There has been no consensus on an ideal lung recruitment strategy, which influences the effect of LRMs. The LRMs’ effects could depend on the pressure level achieved and the duration of exposure. The use of a high PEEP (over 40cmH_2_O) during an LRM is usually accompanied by an increase in transpulmonary pressure, which can increase the risks of barotrauma and morality in ARDS patients [43]. Studies show that a two stepwise inflation of the lung with an airway pressure greater than 30 cm H_2_O and a PEEP of 10-15 cm H_2_O [44] can result in a marked reduction in atelectasis (83%) and effective lung recruitment, consistent with our findings. Moreover, studies have shown that LRMs achieved with a lower pressure could achieve the recruitment goal [45,46] with less circulatory depression and a lower risk of barotrauma [47].

Our study had several limitations: First of all, the sample was relatively small. Second, we did not measure the long-term outcomes. Third, we only presented and analyzed the results of the respiratory parameters and EIT data at the first intervention. Fourth, pulmonary perfusions in patients were not measured in this study, even though they could reflect ventilation–perfusion matching directly.

## 5. Conclusions

In the series of moderate to severe ARDS, PEEP-induced LRMs combined with the prone position acted synergistically to create more beneficial effects, including increased oxygenation and more uniform ventilation distributions. The R/I ratio can be useful to predict the responses to LRMs.

## Figures and Tables

**Table 1:** Baseline characteristics of participants.

	ALL (*n* = 58)	PP Combined LRM (*n* = 28)	PP (*n* = 30)	*p*
**Demographic data**				
Age, mean (SD), years	61.5 (15)	60 (17.5)	64 (12.8)	0.16
Female sex, *n* (%)	11 (18.9%)	4 (14.2)	7 (23.3)	0.38
BMI, median (IQR), kg/m^2^	22.5 (20.7, 25.3)	22.6 (21.1, 26.3)	22.4 (19.8, 24.7)	0.41
APACHE II at ICU admission, median (IQR)	23 (21, 27)	24 (21, 28)	23 (18.27)	0.07
RASS at ICU admission, median (IQR)	−4 (−4.5, −3)	−4(−4, −3)	−4(−4, −3)	0.24
Days intubated prior to randomization, median (IQR)	2(1, 4)	2(1, 3)	2(2, 4)	0.33
**Etiology of ARDS, *n* (%)**				
COVID-19	28 (48.2)	14 (50.0)	14 (46.7)	0.80
Bacterial pneumonia	15 (25.9)	8 (28.6)	7 (23.3)	0.65
Extrapulmonary	15 (25.9)	6 (21.4)	9 (30.0)	0.46
**Comorbidities, *n* (%)**				
Hypertension	24 (41.3)	14 (50.0)	10 (33.3)	0.45
Diabetes mellitus	10 (18.9)	3 (10.7)	7 (23.3)	0.30
Renal insufficiency	27 (46.5)	15 (53.5)	12 (40.0)	0.30
Hepatic insufficiency	22 (37.9)	10 (35.7)	12 (40.0)	0.73
**Hemodynamics, median (IQR)**				
Heart rate (bpm)	98 (78, 116)	100 (82, 125)	90 (76, 115)	0.31
SpO_2_ (%)	99 (97, 100)	98 (96, 100)	99 (98, 100)	0.20
Mean arterial pressure (mmHg)	85 (75.97)	89 (76, 101)	85 (72, 94)	0.15
**Baseline ventilator Settings in supine position, median (IQR)**				
Tidal volume (mL)	449 (411, 492)	445 (410.491)	447 (302, 489)	0.79
Respiratory rate, breaths/min	20 (18, 25)	20 (18, 22)	22 (18, 28)	0.31
PEEP (cmH_2_O)	10 (8, 12)	10 (10, 12)	10 (6, 10)	0.09
C_rs_ (mL/cmH_2_O)	35 (23, 43)	36 (27, 42)	35 (25, 43)	0.37
Ppeak (cmH_2_O)	27 (24, 33)	27 (24, 32)	30 (24, 39)	0.78
Pplat (cmH_2_O)	22 (19, 27)	23 (19, 26)	24 (21, 32)	0.48
Pmean (cmH_2_O)	16 (13, 18)	16 (14, 18)	15 (12, 19)	0.29
Raw(cmH_2_O)	11 (7, 16)	10 (7, 19)	13 (6, 15)	0.93
Driving preassure (cmH_2_O)	11 (5.5, 15.5)	11 (9, 15)	13 (7.5, 16)	0.75
**Arterial blood gas** **, median (IQR)**				
pH	7.35 (7.29, 7.41)	7.34 (7.29, 7.4)	7.37 (7.29, 7.44)	0.38
PaO_2_ (mmHg)	94.7 (80.5, 120.5)	88.8 (75.2, 121.9)	99.1 (82.0, 119.4)	0.45
PaCO_2_ (mmHg)	43.6 (37.8, 52.5)	44 (38.9, 51.3)	42.7 (36.5, 55.3)	0.65
HCO_3_^−^ (mmol/L)	23.9 (21.4, 25.85)	23.2 (20.9, 25.8)	24.3 (21.7, 26.2)	0.49
Lactate (mmol/L)	1.7 (1.3, 2.1)	1.6 (1.2, 2.0)	1.7 (1.4, 2.2)	0.21
Base excess (mmol/L)	−1.2 (−3.7, 1.6)	−1.9 (−4.5, 2)	−0.1 (−3.3, 1.6)	0.71
PaO_2_/FiO_2_ (mmHg)	108 (86, 144)	112 (89, 142)	105 (83, 149)	0.73

ARDS, acute respiratory distress syndrome; BMI, body mass index; APACHE II, Acute Physiology and Chronic Health Evaluation; RASS, Richmond agitation–sedation scale; ICU, intensive care unit; IQR, interquartile range; COVID-19, coronavirus disease 2019; bpm, beats per minute; SpO_2_, pulse oxygen saturation; PEEP, positive end-expiratory pressure; C_rs_, respiratory system compliance; Ppeak, peak pressure; Pplat, plat pressure; Pmean, mean airway pressure; Raw, airway resistance; PaO_2_, partial pressure of arterial oxygen; PaCO_2_, partial pressure of arterial carbon dioxide; HCO_3_^−^, bicarbonate; PaO_2_/FiO_2_, ratio of the partial pressure of arterial oxygen to the fraction of inspired oxygen. Results are reported as median values (interquartile range (IQR)) or number (percentages), as appropriate. *p*-values were obtained by the Student’s *t*-test, Fisher’s exact test, and chi-squared test analysis as appropriate.

**Table 2:** Respiratory and hemodynamic parameters.

Parameters, Median (IQR)	Prone Position Combined with PEEP-Induce LRM (28)				Prone Position (30)			
	T0	T1	T2	*p*	T0	T1	T2	*p*
Resperatory mechanics								
PEEP (cmH_2_O)	8 (6, 10)	8 (6, 10)	8 (6, 10)	0.68	8.5 (7, 10)	8.5 (7, 10)	8.5 (7, 10)	0.39
Ppeak (cmH_2_O)	28.5 (26.25, 34.75)	30.5 (27, 34)	26.5 (24, 31) ^b^ (*p* = 0.01)	0.002 *	26.50 (23, 32.25)	27.5 (22.75, 35.25)	25.0 (22.75, 34)	0.15
Pplat (cmH_2_O)	23.5 (20, 26)	24 (20.25, 25.75)	22.5 (20, 26)	0.38	21.00 (17.75, 26)	22.5 (18, 27.25)	21.0 (17.75, 27.25)	0.45
Pmean (cmH_2_O)	17 (15, 18)	17 (16, 19)	16 (14, 18) ^b^ (*p* = 0.02)	0.02 *	15 (14, 18.25)	16.5 (13, 18)	15 (12.75, 17)	0.04
Tidal volume, (mL)	446.5 (419.3, 489.5)	460.5 (428.3, 502.8)	464 (435, 502.5)	0.24	431.5 (402.8, 491.3)	442.5 (396.8, 487.8)	447 (385.3, 515.8)	0.16
MV (L/min)	9.21 (8.26, 11.23)	10.3 (8.3, 13.1)	9.89 (8.2, 12.5)	0.31	10 (8.28, 11.33)	10.55 (8.57, 11.83)	10.12 (8.09, 11.88)	0.90
C_rs_ (mL/cmH_2_O)	35 (27, 42.75)	35.5 (28.5, 46.25)	35 (28.25, 47)	0.26	30 (25, 43)	32 (22.75, 43)	30 (20, 46)	0.46
Raw (cmH_2_O)	11 (8, 18)	11 (9, 16)	9(7, 11) ^b^ (*p* = 0.02)	0.004 *	11 (6, 18)	12 (4.25, 17.5)	7.55 (4.75, 15.25) ^b^ (*p* = 0.03)	0.06
PaO_2_ (mmHg)	95.4 (81.5, 126.2)	149.1 (120.9, 173.2) ^a^ (*p* < 0.0001)	125.6 (94.2, 166.5) ^b^ (*p* = 0.002)	<0.0001 *	94.7 (75.98, 122.4)	107 (79.48, 131)	113.6 (93.7, 129.9) ^b^ (*p* = 0.04)	0.04
PaCO_2_ (mmHg)	45.05 (40.48, 48.38)	42.7 (40.15, 47.45)	43.3 (39.65, 48.78)	0.53	46.4 (40.1, 52.75)	46.4 (40.7, 52.48)	44.9 (38.7, 51.43)	0.09
PaO_2_/FiO_2_, mmHg	177 (141.6, 194.8)	242.9 (214, 292.5) ^a^ (*p* < 0.0001)	238.3 (176.4, 328.7) ^b^ (*p* < 0.0001)	<0.0001 *	172.3 (122.5, 195.1)	177.8 (116.7, 213.2)	193.3 (122.8, 238.0) ^b^ (*p* = 0.02)	0.03
Hemodynamic parameters								
Heart rate (bpm)	87 (74.25, 98.75)	84 (77.5, 94)	80.5 (69.25, 91)	0.39	85.5 (74.75, 100.3)	80.5 (69.5, 98)	82 (69, 96.25)	0.50
SpO_2_ (%)	100 (98, 100)	99 (98, 99)	100 (99, 100)	0.66	100 (98, 100)	99 (98, 99)	100 (99, 100)	0.83
MAP (mmHg)	97 (82.25, 103.8)	89.5 (76.5, 99.5)	86 (86, 99.25)	0.08	84 (76, 93.5)	85.5 (76.75, 93.75)	81.5 (77, 90)	0.20

LRM, lung recruitment maneuver; Ppeak, peak pressure; Pplat, plat pressure; Pmean, mean airway pressure; MV, minute ventilation volume; C_rs_, respiratory system compliance; Raw, airway resistance; PaO_2_, partial pressure of arterial oxygen; PaCO_2_, partial pressure of arterial carbon dioxide; HCO_3_^−^, bicarbonate; PaO_2_/FiO_2_, ratio of the partial pressure of arterial oxygen to the fraction of inspired oxygen; bpm, beats per minute; SpO_2_, pulse oxygen saturation; MAP, mean arterial pressure. Results are reported as median values (interquartile range (IQR)). Differences between groups, for normally distributed variables, are tested using the one-way repeated measure ANOVA and Tukey post hoc tests for multiple comparisons, while non-normally distributed variables are tested using the non-parametric Kruskal–Wallis test. ^a^ *p* < 0.05 for the ANOVA test was significant between T0 and T1. ^b^ *p* < 0.05 for the ANOVA test was significant between T0 and T2. * *p* < 0.05 for the ANOVA test was significant for repeated measures.

**Table 3:** Mortality among patients in two groups.

	Prone Position Combined LRM (*n* = 28)	PP (*n* = 30)	*p*
**M** **ortality**			
28-day mortality	13 (46.4%)	18 (60.0%)	0.30
90-day mortality	16 (57.1%)	20 (66.6%)	0.46
Overall mortality	19 (67.9%)	22 (73.3%)	0.65
**Length of stay, d**			
Intensive care unit,	22 (17, 38)	16 (9, 25)	0.041 *
Hospital	38 (21, 48)	24 (15, 32)	0.044 *
**Mechanical ventilation, d**	19 (10, 27.5)	15 (9, 23)	0.13
**Adverse event, *n*%**			
Pneumothorax	1 (3.3%)	0	0.48

LRM, lung recruitment maneuver; PP, prone position. Results are reported as median values (interquartile range (IQR)) or number (percentages), as appropriate. Normality of the data distribution was verified using the Shapiro–Wilk test. Differences between the groups were assessed using the Student’s *t*-test or Mann–Whitney rank sum test when appropriate. The chi-squared test of independence was used to test the significance in the case of frequency counts. * *p* < 0.05.

**Table 4:** Comparison of changes in respiratory and EIT parameters with R/I < 0.5 vs. R/I > 0.5, *n* = 28.

	Change in R/I > 0.5 after LRM (*n* = 16)	Change in R/I < 0.5 after LRM (*n* = 12)	*p*-Value	Change in R/I > 0.5 after pp (*n* = 16)	Change in R/I < 0.5 after pp (*n* = 12)	*p*-Value
∆tidal volume, (mL)	10.0 (−10.0, 42.25)	4.5 (−21.75, 57.25)	0.53	−1.0 (−16.5, 19.0)	4.0 (−17.0, 38.0)	0.36
∆MV (L/min)	0.1 (−0.76, 1.13)	0.0 (−0.61, 2.2)	0.50	−0.1 (−0.67, 0.47)	−0.03 (−0.65, 0.99)	0.39
∆C_rs_ (mL/cmH_2_O)	3.5 (0.25, 7.0)	1.0 (−6.25, 4.0)	0.18	2.5 (−1.5, 10.3)	-0.5 (−3.0, 9.5)	0.55
∆PaO_2_ (mmHg)	76.05 (28.95, 117.9)	21.45 (12.23, 43.4)	0.01 *	90.85 (14.05, 125.3)	50.3 (21.2, 79.2)	0.53
∆PaO_2_/FiO_2_ (mmHg)	140.5 (55.4, 170.6)	47.6 (25.85, 87.2)	0.02 *	51.65 (15.7, 161.8)	54.85 (24.55, 144.9)	0.83
***EIT data***						
∆TV ROI 1 layers (%)	−2.0 (−3.75, −1.0)	−0.5 (−2.75, 0.0)	0.21	−1.0 (−11.25, 7.5)	−5.5 (−14.75, −0.25)	0.28
∆TV ROI 2 layers (%)	−4.0 (−7.0, 1.0)	0.0 (−4.0, 2.0)	0.14	4.0 (−0.75, 12.75)	4.0 (−6.75, 6.75)	0.32
∆TV ROI 3 layers (%)	5.0 (0.75, 6.75)	2.0 (1.0, 3.75)	0.02 *	−6.0 (−15.25, 6.75)	2.0 (−10.25, 8.25)	0.28
∆TV ROI 4 layers (%)	1.0 (0.25, 4.0)	0.5 (−0.75, 2.0)	0.26	2.0 (−1.75, 4.0)	5.5 (0.25, 6.75)	0.99
∆ventral of tidal image region (%)	−6.5 (−9.0, −0.25)	−2.5 (−4.75, 1.25)	0.07	4.0 (−9.25, 13.5)	−5.5 (−12.75, 6.25)	0.10
∆dorsal of tidal image region (%)	6.0 (1.5, 9.0)	4.0 (−0.75, 4.75)	0.04 *	−2.5 (−11.25, 11.0)	4.0 (−5.0, 10.5)	0.37

R/I ratio, recruitment-to-inflation ratio; LRM, lung recruitment maneuver; PP, prone position; MV, minute ventilation volume; C_rs_, respiratory system compliance; PaO_2_, partial pressure of arterial oxygen; PaO_2_/FiO_2_, ratio of the partial pressure of arterial oxygen to the fraction of inspired oxygen; EIT, electric impedance tomography; TV, tidal volume, ROI, region of interest. Results are reported as median values (interquartile range (IQR)). Normality of data distribution was verified with the Shapiro–Wilk test. Differences between groups was assessed with the Student’s *t*-test or Mann–Whitney rank sum test when appropriate. * *p* < 0.05.

## Data Availability

All the data analyzed and discussed in the framework of this study are included in this published article and its online Supplementary Materials.

## Supplementary Materials

The following supporting information can be downloaded at: https://www.mdpi.com/article/10.3390/jcm13030853/s1, Ref. [48] is cited in supplementary files.

## Abbreviations

PEEP: positive end-expiratory pressure; ARDS, acute respiratory distress syndrome; EIT, electrical impedance tomography; LRM, lung recruitment maneuver; ICU, intensive care unit; COPD, chronic obstructive pulmonary disease; R/I ratio, recruitment-to-inflation ratio; PaO_2_/FiO_2_, ratio of the partial pressure of arterial oxygen to the fraction of inspired oxygen; COVID-19, coronavirus disease 2019; VILI, ventilator-induced lung injury; ALI, acute lung injury; Vt, tidal volume; TV, tidal volume; CRP, C-reactive protein; APACHE II, Acute Physiology and Chronic Health Evaluation; RASS, Richmond agitation–sedation scale; ABG, arterial blood gas; ROI, region of interest; TIV, tidal impedance variation; EELV, end-expiratory lung volume; Vrec, recruitment volume; Crec, recruitment compliance; AOP, airway opening pressure; Crs, respiratory system compliance; IQR, interquartile range; PP, prone position; SD, standard deviation; BMI, body mass index; SpO_2_, oxygen saturation as measured by pulse oximetry; bpm, beats per minute; P_peak_, peak pressure; P_plat_, plat pressure; P_mean_, mean airway pressure; R_aw_, airway resistance; PaO_2_, partial pressure of arterial oxygen; PaCO_2_, partial pressure of arterial carbon dioxide; HCO_3_^−,^ bicarbonate; MV, minute ventilation volume; CT, computed tomography; MAP, mean arterial pressure.
