# Supplementary material for: PEEP-Induced Lung Recruitment Maneuver Combined with Prone Position for ARDS: A Single-Center, Prospective, Randomized Clinical Trial
Source: J Clin Med. 2024 Feb 1;13(3):853. doi: 10.3390/jcm13030853 (PMC10856548; doi:10.3390/jcm13030853)

SUPPLEMENTARY Appendix

List of contents

APPENDIX S1: Methodology………………………………………………………...2

APPENDIX S2: Supplemental table……………………………………………….....4

APPENDIX S3: Supplemental figure ……………………………………………..20

Methodology

S1. Mechanical ventilation settings

All patients were ventilated in a volume-controlled mode. The ventilator settings were standardized for all patients during all study measures that followed the lung-protective ventilation principles. Principle of the parameter setting:

(1) Tidal volume (Vt): 6–8mL/kg of predicted body weight. Constant inspiratory flow: 50-60L/min. Predicted body weight to be calculated for all patients according to the NHLBI ARDS network formula [1]:

Men: Predicted body weight（Kg）=50+2.3((height[cm]*0.394)-60)

Women: Predicted body weight（Kg）=45.5+2.3((height[cm]*0.394)-60)

(2) Respiratory rate: set the respiratory rate to maintain a minute ventilation similar to that recorded before the study enrollment.

(3) PEEP and FiO_2: select the optimal initial levels of PEEP and FiO_2 to maintain the oxygenation target.

[1] Acute Respiratory Distress Syndrome Network; Brower RG, Matthay MA, Morris A, Schoenfeld D, Thompson BT, Wheeler A. Ventilation with lower tidal volumes as compared with traditional tidal volumes for acute lung injury and the acute respiratory distress syndrome. N Engl J Med. 2000 May 4;342(18):1301-8. Doi: 10.1056/NEJM200005043421801. PMID: 10793162.

S2. Prone position

Clinicians evaluated the patient's condition and decided whether the prone position should be applied. The time of applying the prone position was settled at 12 hours per day (24 hours) both for the control group and intervention group. The EIT, ABG, and respiratory mechanics were recorded 1 hour before the prone position, 1 hour after the end of the lung recruitment, and 1 hour after finishing the prone position. The airway was cleared before the prone position started, and the treatment was carried out according to the routine analgesia and sedation process. The goal of analgesia is to maintain a CPOT score of 0, while the sedation standard is based on the RASS score (<-2). A total of 4-6 medical staff underwent postural conversions and a PEEP-induced lung

recruitment was performed 1 hour after the initiation of the prone position when the hemodynamics and other parameters were stable.

S3. Peep-induced lung recruitment maneuver

Firstly, patients were ventilated in a volume-controlled mode with a VT at 6 ml/kg of the predicted body weight. Peep was set after a baseline assessment by the clinicians. There were three steps in the peep-induced lung recruitment: (1) PEEPhigh, maintained for 30 minutes; then, PEEPhigh was adjusted to PEEPlow by a prolonged deflation maneuver. (2) PEEPlow, maintained 30 minutes. (3) PEEPhigh was repeated and maintained for 30 minutes. Then, we restored the initial parameters for the invasive ventilation. FiO2 and other settings were planned to be kept unchanged during the study. The procedures for the prolonged deflation maneuver were noted: 1) reduce the respiratory frequency (RR) to 6-8 breaths per minute to allow a prolonged expiration and 2) after one or two breaths, reduce the PEEP from PEEP high to PEEP low. In the intervention group, the EIT assessment and other respiratory parameters were recorded in the supine position (T0) at the end of the PEEP-induced LRM (T1) and at the end of prone position (T2). In the control group, the EIT assessment and other respiratory parameters were recorded in the supine position (T0), 3 hours after the prone position initiation (T1), and at the end of the prone position (T2). Each patient in the intervention group received a peep-induced lung recruitment maneuver once per day within 3 days.

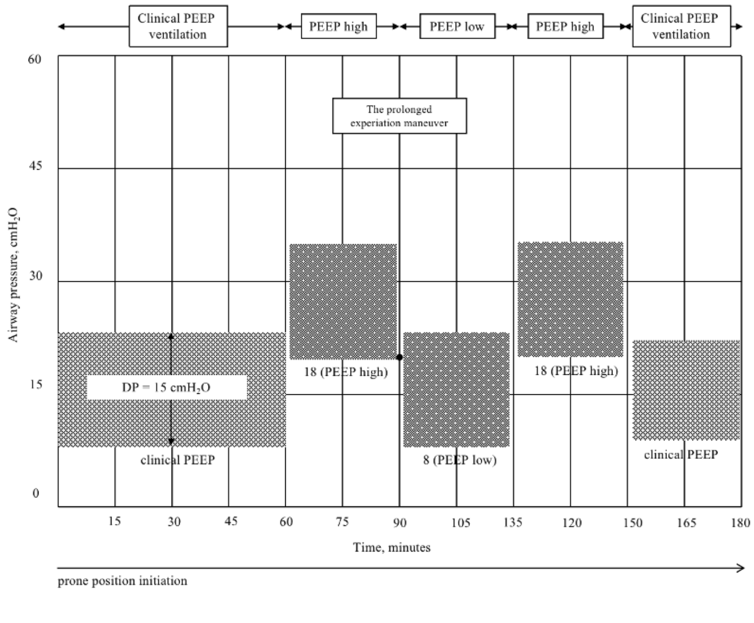

Supplementary Figure S4. Protocol for the peep-induce lung recruitment in patients.

S4. Other treatments

While the patients stayed in the hospital, the clinicians managed the patients according to the best clinical practice evidence, evaluated and dealt with the risk factors in time to prevent aggravating hypoxia, such as anti-infection, optimized fluid therapy, maintained appropriate hemoglobin levels, improved the balance of oxygen supply and demand, and prevented complications.

S5. Sample size estimation

The sample size calculation was based on the consideration that a 10% change in the regional ventilation was clinically relevant. A total of 13 patients in each group were required, assuming a standard deviation of a change of 10%, a power of 0.9, and alpha level of 0.05. Our small sample size represented a convenience sample similar in scope to other EIT studies.

S6. The periods of recruitment and follow ups

Our trial was registered on 27 June 2023, which was prospectively registered. The enrolment was between June and December 2023.

Supplemental table

Supplementary Table S1. Characteristics of laboratory and clinical data for patients included.

	ALL (n=58)	PP Combined LRM (n=28)	PP (n=30)	P
Laboratory and clinical data				
C-reactive protein (mg/L)	125 [71.9, 225.5]	143[80,320]	125.5[62.6,220.5]	0.51
procalcitonin (ng/L)	1.47[0.46, 4.95]	2.26[0.79,11.9]	0.9[0.3,3.8]	0.25
interleukin-6 (pg/L)	114.5[37.1, 323.8]	124.3[37.7, 237.6]	89.5[32.5, 603.5]	0.60
D-dimer (μg/l)	6.2[2,14.9]	6.2[2,17.6]	9.5[2.1,15.2]	0.47
Brain natriuretic peptide (ng/L)	1659[729,4110]	1785[807,2901]	1646[636,5474]	0.52
Absolute value of neutrophils (10*9/L)	9.2[5.91,12.73]	8.7[5.4,13]	9.8[6.6,12.7]	0.77
AST(U/L)	36 [24.5,52.5]	23[16,24]	25 [17,54]	0.63
ALT(U/L)	25 [17,43]	32 [22,43]	36 [25,56]	0.29
LDH(U/L)	409 [299.5,575.5]	477[352,662]	369[287,454]	0.93
Albumin (g/L)	29.7 [26.25,31.95]	29.6 [26.3,32.2]	30.3 [26.1,32.3]	0.52
Total bilirubin (umol/L)	10 [5.55,15.95]	10.1 [5,15.5]	9.8 [5.7,16.4]	0.95
Creatine kinase	1.93[1.02,2.82]	1.97 [0.84,3.01]	1.76 [1.08,2.78]	0.85
Urea (mmol/L)	12.7[7.65,19.55]	14.6[11.68,23.23]	10.1[6.65,16.02]	0.10

Na^+(mmol/l)	140.7[136.7,144.7]	141[137.5,144.8]	140.8 [136.5,146.3]	0.88
K^+(mmol/l)	4.46[3.79,4.84]	4.5 [3.59,5.06]	4.45 [3.68,4.7]	0.71
Cl^-(mmol/l)	104.4[102.1,110.6]	104.8[101.3,110.6]	108.4 [102.4,110.9]	0.89
leucocyte(10^{*9}/L)	9.94[6.99,14.76]	10.8[5.66,15.29]	9.66 [6.87,14.97]	0.56

Baseline laboratory and clinical data. There were no significant differences in the characteristics of laboratory and clinical data listed between the two groups. LRM, lung recruitment maneuver; PP, prone position; AST, aspartate aminotransferase; ALT, alanine transaminase; LDH, lactate dehydrogenase; Na^+, sodium ion; K^+, potassium ion; Cl^-, chloride ion. Results are reported as median values (interquartile range (IQR)). *p*-values were obtained from Student's t-test and Wilcoxon's test as appropriate.

Supplementary Table S2. Trend of respiratory mechanics and oxygenation parameters or 3 consecutive interventions/days in the PP combined with LRM group and PP group.

**Parameters**	Prone Position Combined with LRM				Prone Position			
	Day 1	Day 2	Day 3	*p*-value	Day 1	Day 2	Day 3	*p*-value
Rass (median [IQR])	-4[-4,-4]	-4[-4,-4]	-4[-4,-4]	0.26	-4[-4,-4]	-4[-4,-4]	-4[-5,-4]	0.69
Respiratory mechanics								
Ppeak(cmH_2O)	28.5[26.25,34.75]	26.5[24,31][a] (p=0.01)	23[21,26][b] (p=0.001)	0.001*	26 [24,35.5]	25 [22.75,30.5][a] (p=0.04)	25[24,31]	0.02*
Pplat(cmH_2O)	23 [19.25,26]	21.5[20,25.75]	21 [17.75,24]	0.06	21 [18,29.25]	19.5[18,26.25]	20[18.5,28]	0.22
Pmean(cmH_2O)	17 [15,18]	16 [14.25,18]	15.5[14,18]	0.04*	16[12.75,18.25]	15[13.75,17]	14[13,16]	0.10
Tidal volume (ml)	450.5[412.5,492.5]	449.5[392,497.8]	448[413.5,494.3]	0.89	447[401.3,489.8]	442[395.3,506.5]	436[401,513]	0.69
MV(L/min)	9.87[8.51,11.23]	9.49[8.24,10.98]	9.03[8.18,11.28]	0.37	9.87[7.95,11.63]	10.1[8.04,11.25]	9.54[8.8,10.9]	0.86
C_{rs}(ml/cmH_2O)	35 [27,42]]	39.5[29.75,44.75]	39[34.75,50][b] (p=0.03)	0.10	28.5[22.75,42.25]	31.5[22.75,49.25]	35[26.25,45]	0.22

Raw(cmH_2O)	9 [6.25,13.5]	9.5 [7,11.75]	8 [6,9]	0.06	10[6.83,14.25]	8.9[5,13]	7.65[4.25,14.5]	0.11
PaO_2(mmHg)	95.4[81.5,126.2]	115.4[96.83,137.5] [a](p=0.04)	119.3[105.5,133.1][b] (p=0.01)	0.01*	94.7[75.98,122.4]	110.6[89.03,130.6]	116.5[94.75,125.9]	0.08
$PaCO_2$(mmHg)	45.05[40.48,48.3 8]	43.55[38.33,47.15]	42.8[38.93,53.08]	0.31	46.4[40.1,52.75]	45.7[43.15,53.43]	43.6[39.65,49.95]	0.65
HCO_3-(mmol/L)	24.35[22.8,27.8]	25.4[24.05,29.23]	26.5[23.73,30.0][b] (p=0.03)	0.03	26.8[24.8,31.5]	28.1[24.25,24.25]	29.6[25.25,31.2]	0.14

Trend of respiratory mechanics monitoring for 3 consecutive interventions/days in prone position combined with LRM group and prone position group.

LRM, lung recruitment maneuver; Ppeak, peak pressure; Pplat, plat pressure; Pmean, mean airway pressure; MV, minute ventilation volume; C_{rs}, respiratory system compliance; Raw, airway resistance; PaO_2, partial pressure of arterial oxygen; $PaCO_2$, partial pressure of arterial carbon dioxide; HCO_3-, bicarbonate. Results are reported as median values (interquartile range (IQR)). Differences between groups, for normally distributed variables, are tested with one-way repeated measure ANOVA and Tukey post hoc tests for multiple comparisons, while non-normally distributed variables are tested with the non-parametric Kruskal–Wallis test.

a: $p<0.05$ for ANOVA test significant between days 1 and 2.

b: $p<0.05$ for ANOVA test significant between days 1 and 3.

*: $p < 0.05$ for the ANOVA test significant for repeated measures.

Supplementary Table S3. Comparison of changes in ventilator parameters with PP combined with LRM vs. PP; for all participants, n=58

Respiratory mechanics	Change in PP combined with LRM after 3 days	Change in pp after 3 days	*p*-value
ΔPpeak(cmH_2O)	-2 [-5,2]	-2 [-5.25,1]	0.84
ΔPplat(cmH_2O)	-2 [-4,0]	-1 [-4.5,2]	0.48
ΔPmean(cmH_2O)	0 [-2,0]	-1 [-2,0.5]	0.70
Δtidal volume (ml)	4 [-44,43.75]	2 [-37,22.5]	0.96
ΔMV(L/min)	-0.22 [-1.22,0.69]	-0.2 [-0.85,1.29]	0.64
ΔC_{rs}(ml/cmH_2O)	4.5 [0,13.25]	1.29[-2.75,8]	0.19
ΔRaw(cmH_2O)	-2 [-4.25,1]	-1.85[-6,0.75]	0.72
ΔPaO_2(mmHg)	14.35[-5.15,38.1]	11 [0.25,26.3]	0.48
Δ$PaCO_2$(mmHg)	-1.4[-6.45,5.2]	-1.9 [-8.6,4.1]	0.67
ΔHCO_3-(mmol/L)	2.1 [0.3,3.7]	1.7 [-1.3,4.8]	0.99
ΔPaO_2/FiO_2(mmHg)	92.45[60.55,151.7]	61.75[19.83,114.2]	0.04*

Comparison of changes in respiratory mechanics between PP combined with LRM and PP groups after 3 consecutive interventions/days.
LRM, lung recruitment maneuver; PP, prone position; Ppeak, peak pressure; Pplat, plat pressure; Pmean, mean airway pressure; MV, minute ventilation volume; C_{rs}, respiratory system compliance; Raw, airway resistance; PaO_2, partial pressure of arterial oxygen; $PaCO_2$, partial pressure of arterial carbon dioxide; HCO_3-, bicarbonate; PaO_2/FiO_2, ratio of the partial pressure of arterial oxygen to the fraction of inspired oxygen. Results are reported as median values (interquartile range (IQR)). Normality of data distribution was verified with the Shapiro–Wilk test. Differences between groups were assessed with Student’s t-test or Mann–Whitney rank sum test when appropriate. *P<0.05.

Supplementary Table S4. Comparison of changes in ventilator parameters with PP combined with LRM vs. PP; for all participants, n=58

Respiratory mechanics	Change in PP combined with LRM after LRM	Change in pp after 3 hours	*p*-value	Change in PP combined with LRM After pp	Change in pp After pp	*p*-value
ΔPpeak(cmH_2O)	0.5 [-1,3]	0 [-2,4]	0.86	-4 [-5.75,2]	-0.5 [-5.25,2]	0.34
ΔPplat(cmH_2O)	-0.5 [-1,2]	1 [-1,2]	0.41	-0.5 [-3,1]	-0.5 [-2,1.25]	0.56
ΔPmean(cmH_2O)	0 [-1,1]	0 [-1,1.25]	0.71	-1 [-1,0]	-0.5 [-2.25,0.25]	0.96
Δtidal volume (ml)	7 [-16,42.25]	1 [-22.75,10.25]	0.15	2 [-15.75,25.25]	1.5 [-14,28]	0.85
ΔMV(L/min)	0.1 [-0.64,1.26]	-0.05[-0.77,1.01]	0.64	-0.09[-0.68,0.5]	-0.01[-0.61,0.73]	0.96
ΔC_{rs}(ml/cmH_2O)	2 [-3,5.75]	-1 [-3.25,0.5]	0.02*	1.5 [-2.75,9.5]	-1 [-5,3.25]	0.14
ΔRaw(cmH_2O)	0 [-3,2.5]	-1 [-4.8,2]	0.37	-2 [-4.5,0.25]	-1.75[-7,1]	0.81
ΔPaO_2(mmHg)	44.15[21.13,84.95]	6.3 [-2.9,22.68]	<0.0001*	18.1[0.8,59.28]	13.1[-7.08,28.6]	0.17
Δ$PaCO_2$(mmHg)	-0.45[-4.78,2.38]	0.4 [-1.6,4.58]	0.45	-0.85 [-4.75,2.58]	-1.5[-7.03,1.8]	0.63
ΔHCO_3-(mmol/L)	-0.15 [-0.8,1.05]	0.55[-1.05,1.93]	0.22	0.2 [-1.13,1.68]	0.8 [-0.93,2.53]	0.46
ΔPaO_2/FiO_2(mmHg)	71.15[40.1,150.8]	4.75[-9.28,4.75]	<0.0001*	51.65[20.2,155.4]	18.5[-6.8,63.75]	0.01*

LRM, lung recruitment maneuver; PP, prone position, Ppeak: peak pressure; Pplat, plat pressure; Pmean, mean airway pressure; MV, minute ventilation volume; C_{rs}, respiratory system compliance; Raw, airway resistance; PaO_2, partial pressure of arterial oxygen; $PaCO_2$, partial pressure of arterial carbon dioxide; HCO_3-, bicarbonate; PaO_2/FiO_2, ratio of the partial pressure of

arterial oxygen to the fraction of inspired oxygen. Results are reported as median values (interquartile range (IQR)). Normality of data distribution was verified with the Shapiro–Wilk test. Differences between groups were assessed with Student's t-test or Mann–Whitney rank sum test when appropriate. *P<0.05.

Supplementary Table S5. Subgroup analysis of COVID-19- and non-COVID-19-associated ARDS.

	COVID-19-associated ARDS			Non-COVID-19-associated ARDS		
	Change after LRM in intervention group (n=14)	Change after LRM in control group (n=14)	*p*-value	Change after LRM in intervention group (n=14)	Change after LRM in control group (n=16)	*p*-value
Tidal volume (ml)	-0.5 [-12,11.25]	0.5 [-12.75,23.75]	0.62	32 [-23,73.25]	1.5 [-63.75,8.5]	0.77
MV(L/min)	0 [-0.8,0.31]	0.05 [-0.13,1]	0.11	0.3[-0.28,2.48]	-0.12 [-0.81,0.58]	0.16
C_{rs}(ml/cmH_2O)	4[-1,7]	-2[-6.25,-0.75]	0.02*	2 [-7.25,4]	0 [-2,5]	0.76
PaO_2(mmHg)	46.9[19.28,84.65]	3.35[-10.88,27.2]	0.004*	31.85[19.05,91.6]	8.8 [-1.83,20.25]	0.012*
PaO_2/FiO_2(mmHg)	76.8[52.25,149.9]	4.6[-12.18,34.83]	<0.001*	54.05[32.95,152.2]	9.9 [-8.53,30.2]	0.006*
EIT data						
TV ROI 1 layers (%)	-2 [-4.25,0]	-3 [-7.5,3]	0.66	-1 [-2.25,0]	-0.5 [-2.75,1.75]	0.91
TV ROI 2 layers(%)	1 [-3,2.25]	2 [-1.25,4.75]	0.33	-1 [-4.25,2]	-0.5 [-2.75,2.75]	0.44
TV ROI 3 layers(%)	4.5 [0.5,6]	2 [-0.5,4.25]	0.25	3.5 [0.75,4.75]	1 [-2.25,4.5]	0.12
TV ROI 4 layers(%)	2 [1.75,4]	1 [-1,2]	0.045*	1 [-0.25,4]	0 [0,1]	0.57
Ventral of tidal image region (%)	-2.5[-6.5,2.25]	-2 [-4.5,1]	0.9	-2.5[-4.25,0.25]	-1.5 [-4.5,2.5]	0.51
Dorsal of tidal image region (%)	6.5 [1,9.25]	3 [0,5]	0.03*	5 [3.5,8]	2 [0,4.5]	0.07

COVID-19, coronavirus disease 2019; ARDS, acute respiratory distress syndrome; LRM, lung recruitment maneuver; MV, minute ventilation volume; C_{rs}, respiratory system compliance; PaO_2, partial pressure of arterial oxygen; PaO_2/FiO_2, ratio of the partial pressure of arterial oxygen to the fraction of inspired oxygen; EIT, electric impedance tomography; TV, tidal volume, ROI, region of interest. Results are reported as median values (interquartile range (IQR)). Differences between groups were assessed with Student's t-test or Mann–Whitney rank sum test when appropriate. *P<0.05

Supplementary Table S6. EIT data measurements at three time points between two groups.

**Parameters**	Prone Position Combined with LRM				Prone position			
n%	T0	T1	T2	P	T0	T1	T2	P
RR	21.61(4.85)	22.11(4.34)	21.54(5.21)	0.60	20.97（4.97）	20.23（5.32）	22.4（5.62）	0.11
EIT layers								
TV ROI 1	16.57(9.94)	15.27(9.43)[a](P=0.03）	13.33(5.21)	0.07	15.88(9.37)	13.88(7.67)[a](p=0.04)	13.84(6.39)	0.10
TV ROI 2	37.43(6.63)	37.03(7.24)	39.33(6.84)	0.16	41.22(6.71)	42.5(7.35)	40.88(7.3)	0.19
TV ROI 3	35.3(9.99)	38.67(10.03)[a](P<0.0001)	35.83(8.35)	0.05	33.22(10.09)	35.06(8.9)	34.97(8.83)	0.48
TV ROI 4	8.43(3.16)	10.17(3.23)[a](p=0.003)	13.10(4.82)[b](<0.001)	<0.0001*	8.75(5.17)	9.41(5.58)	10.81(10.81)[b](p=0.01)	0.01*
EIT quadrants								
TV ROI 1	29.26(8.91)	27.37(8.94)	28.52(6.62)	0.21	33.9(11.26)	32.03(10.86)	28.63(10.53)[b](P=0.03)	0.11
TV ROI 2	29.22(8.83)	25.15(9.32)	24.15(8.92)	0.06	25.5(6.76)	23.91(9.13)	22.16(8.99)	0.13
TV ROI 3	23.47(7.66)	25.80(7.77)[a](p=0.001)	24.21(5.95)	0.13	21.66(21.66)	23.67(9.22)[a](p=0.030)	25.53(7.64)	0.07

TV ROI 4	21.25(6.63)	22.64(7.46)	22.11(7.37)	0.57	19.87(7.57)	20 (8.25)	20.03(7.25)	0.96
Ventral of tidal image Region (%)	53.07(10.78)	50.89(11.51)	51.25(8.04)	0.34	57.23(11.27)	55.9(9.9)	55.07(9.88)	0.40
Dorsal of tidal image Region (%)	44.5(10.94)	49.68(11.42)[a](P<0.0001)	49.96(8.72)[b](P =0.001)	0.0001*	41.37(11.2)	43.57(9.9)[a](p=0. 004)	45.3(9.4)[b](p=0 .01)	0.04*

PP, prone position; LRM, lung recruitment maneuver; EIT, electric impedance tomography; TV, tidal volume; ROI, region of interest; T0, in the supine position; T1, 3 h after PP initiation /after LRM; T2, at the end of PP. Results are reported as means ± standard deviations. Normality of data distribution was verified with the Shapiro–Wilk test. Differences between groups were assessed with Student's t-test or Mann–Whitney rank sum test when appropriate. *P<0.05.

a. p<0.05 for ANOVA test significant between T0 and T1.

b. p<0.05 for ANOVA test significant between T0 and T2.

Supplementary Table S7. Comparison of changes in ventilation distribution with PP combined with LRM vs. PP, for all participants.

**Parameters**	Change in PP combined with LRM after LRM	Change in pp after 3 hours	*p*-value	Change in PP combined with LRM after pp	Change in pp after pp	*p*-value
EIT layers						
ΔTV ROI 1	-1.5 [-3,0]	-1.5 [-6,2]	0.86	-3.5 [-11.25,5.25]	-1 [-7.25,3.25]	0.48
ΔTV ROI 2	0.5 [-3.75,2]	0 [-2,3]	0.29	3 [-3,5]	0 [-8.25,5]	0.29
ΔTV ROI 3	4 [1,6]	2 [0,5]	0.19	1.5 [-6,8.25]	1.5[-2.25,5.25]	0.80
ΔTV ROI 4	2 [1,4]	0.5 [-0.25,1.25]	0.03*	4.5[2,7]	2.5 [-0.25,5]	0.03*
EIT quadrants						
ΔTV ROI 1	-4 [-6,0]	-1 [-4.25,1.25]	0.18	-0.5 [-11.25,6]	-5 [-12.25,0.25]	0.20
ΔTV ROI 2	-1 [-3,1.75]	-0.5 [-4.25,1]	0.80	1 [-7.75,8]	-2.5 [-9.25,5.25]	0.19
ΔTV ROI 3	3 [0.25,4]	1.5 [-0.25,5.25]	0.68	-1 [-4,6]	3 [-1.5,9]	0.14
ΔTV ROI 4	1 [-1,3]	0 [-2,3]	0.17	2.5 [-6,6]	1 [-5.25,6]	0.64
Δventral of tidal Image region (%)	-2.5 [-4.75,2]	1 [-2,1]	0.65	-2.5 [-10.75,5]	-3 [-8,3.5]	0.76
Δdorsal of tidal Image region (%)	5 [1.25,8]	2 [0,5]	0.02*	8 [-2,11]	4 [-1.25,8]	0.25

PP, prone position; LRM, lung recruitment maneuver; EIT, electric impedance tomography; TV, tidal volume; ROI, region of interest. Results are reported as median values (interquartile range (IQR)). Normality of data distribution was verified with the Shapiro–Wilk test. Differences between groups were assessed with Student's t-test or Mann–Whitney rank sum test when appropriate. *P<0.05.

Supplementary Table S8. Effects of the prone position combined with the lung recruitment maneuver vs. the prone group on 28-day mortality according to subgroups.

Subgroups	Prone Position Combined with LRM (n=28)	PP (n=30)	Hazard Ratio	HR Absolute Difference (95% CI)	P-interaction
Type of ARDS					0.63
COVID-19	14(50%)	14(46.7%)	0.34	[0.12,1.01]	
Non-COVID-19-related	14(50%)	16(54.3%)	0.91	[0.33,2.52]	
PaO_2/FiO_2					0.29
≤100 mmHg	12(42.8%)	15(50.0%)	0.43	[0.13, 1.47]	
>100 mmHg	16(57.1%)	15(50.0%)	0.51	[0.20,1.28]	
Age					0.12
≤ 65 years	18(64.0%)	13(43.3%)	0.91	[0.29,2.86]	
>65 years	10(36.0%)	17(56.7%)	0.78	[0.27,2.26]	

LRM, lung recruitment maneuver; PP, prone position; HR, hazard ratio; ARDS, acute respiratory distress syndrome; COVID-19, coronavirus disease 2019; PaO_2/FiO_2, ratio of the partial pressure of arterial oxygen to the fraction of inspired oxygen.

R/I Ratio

Supplementary Table S9. Basic characteristics of patients in two groups (R/I >0.5 vs. R/I <0.5).

	R/I >0.5 (n=16)	R/I <0.5 (n=12)	P
R/I ratio	0.78[0.69,0.95]	0.33[0.25,0.44]	<0.001*
AOP(cmH_2O)	4.5[3,7]	5[4,6]	0.58
V_{rec} (ml)	608[525,721]	444[384,541]	0.01*
C_{rs} at PEEP high1(ml/cmH_2O)	31[26,40.7]	24.5[21,37]	0.23
C_{rs} at PEEP low(ml/cmH_2O)	40.5[31.7,44.75]	34[27,48]	0.61
HR at PEEP high1(bpm)	81[70.91]	85[82.91]	0.52
HR at PEEP low(bpm)	88[80,102]	83[78,90]	0.57
MAP at PEEP high1 (mmHg)	92[79,104]	88[69,98]	0.19
MAP at PEEP low (mmHg)	96[80,108]	85[80,98]	0.08

R/I ratio, recruitment to inflation ratio; AOP, airway opening pressure; V_{rec}, recruited volume; C_{rs}, respiratory system compliance; PEEP, positive end-expiratory pressure; HR, heart rate; bpm, beats per minute; MAP, mean arterial pressure. Results are reported as median values (interquartile range (IQR)). Normality of data distribution was verified with the Shapiro–Wilk test. Differences between groups were assessed with Student's t-test or Mann–Whitney rank sum test when appropriate. *P<0.05.

Supplementary Table S10. Physiologic variables at the three different time points.

	R/I>0.5				R/I<0.5			
	T0	T1	T2	P	T0	T1	T2	P
Respiratory Mechanics								
Tidal volume (ml)	466[436.515]	473.5[429.5,429.5]	471[435,91.8]	0.25	430[391,470]	447[420,496]	460[429,528]	0.19
MV(L/min)	10[8.3,11,2]	10[8.4,13]	9.95[8.2,12,3]	0.57	9[8,11.7]	11[7.8,13.1]	9.7[7.8,12.8]	0.46
C_{rs}(ml/cmH_2O)	36[27,41.5]	35.5[33,46]	35[29,46]	0.17	32.5[26,2,46.2]	33.5[25.5,48.5]	38[24.2,50.7]	0.70
PaO_2(mmHg)	96.3[75,130]	162.7[145.4,208.3][a](P<0.01)	130[88.3,182][b](P=0.03)	<0.001*	94[86,122]	126.6[107.4,146.4][a](P<0.01)	119.4[103,148.3][b] (P=0.029)	0.004*
$PaCO_2$(mmHg)	45[40.5,48.4]	42.2[40.1,47.5]	45.5[38.7,48.8]	0.58	44[39,50]	43[41,52.6]	42.4[40.48.8]	0.52
HCO_3-(mmol/L)	25.5[23.3,28.9]	25.4[23.6,27.6]	25.5[22.5,28.7]	0.80	24[22.3,27.6]	24.6[22.9,27.3]	24.4[23.2,26.9]	0.57
PaO_2/FiO_2(mmHg)	169.5[128,194]	265[225,335][a](P<0.01)	236.4[176.4,321][b](P=0.01)	<0.001*	188[159,195]	242[158,260][a](P<0.01)	238[175,326][b](P=0.01)	0.004*
Hemodynamic parameters								

HR (bpm)	88[69,107]	83[69,97]	77[69,93]	0.28	84[78,97]	84[80,93]	85[75,91]	0.08
SpO_2(%)	100[98,100]	99[98.99]	100[99,100]	0.98	99[98,100]	99[98.99]	100[99,100]	0.88
MAP (mmHg)	97[88,105]	95[77,105]	83[79,98]	0.06	93[80,105]	85[76,95]	81[70,100]	0.10

R/I ratio, recruitment to inflation ratio; MV, minute ventilation volume; C_{rs}, respiratory system compliance; PaO_2, partial pressure of arterial oxygen; $PaCO_2$, partial pressure of arterial carbon dioxide; HCO_3-, bicarbonate; PaO_2/FiO_2, ratio of the partial pressure of arterial oxygen to the fraction of inspired oxygen; HR, heart rate; bpm, beats per minute; SpO_2, pulse oxygen saturation; MAP, mean arterial pressure. Results are reported as median values (interquartile range (IQR)). Differences between groups, for normally distributed variables, are tested with one-way repeated measure ANOVA and Tukey post hoc tests for multiple comparisons, while non-normally distributed variables are tested with the non-parametric Kruskal–Wallis test.

a. $p<0.05$ for ANOVA test significant between T0 and T1.

b. $p<0.05$ for ANOVA test significant between T0 and T2.

*: $p < 0.05$ for the ANOVA test significant for repeated measures.

Supplemental figure

Supplementary Figure S1. Flowchart of patients in the ICU for trial.

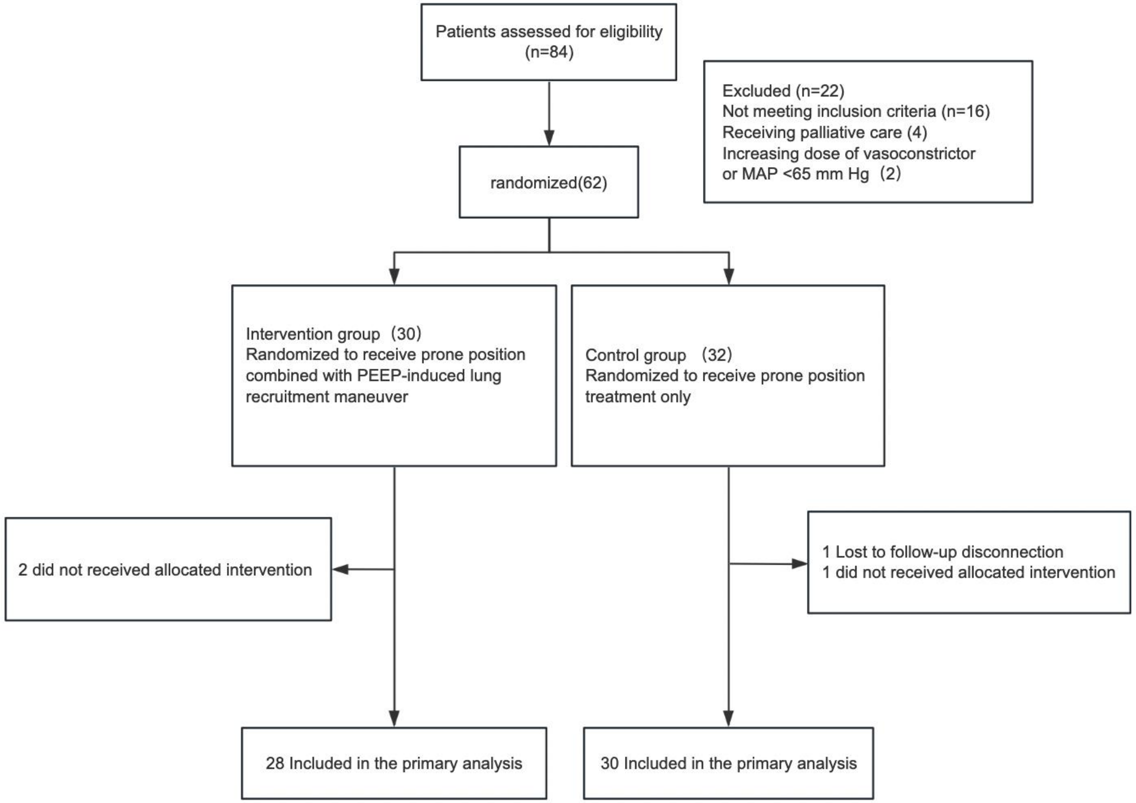

Supplementary figure S2. Consort flow diagram of the trial.

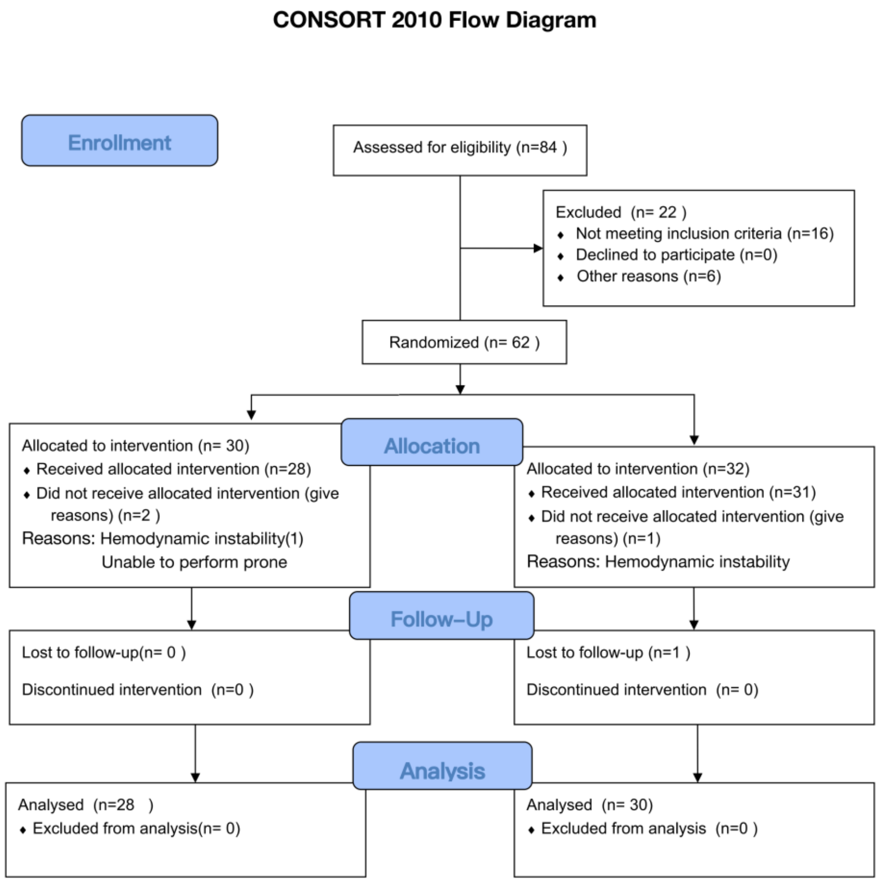

Supplementary figure S3. Flowchart of patients included in trial.

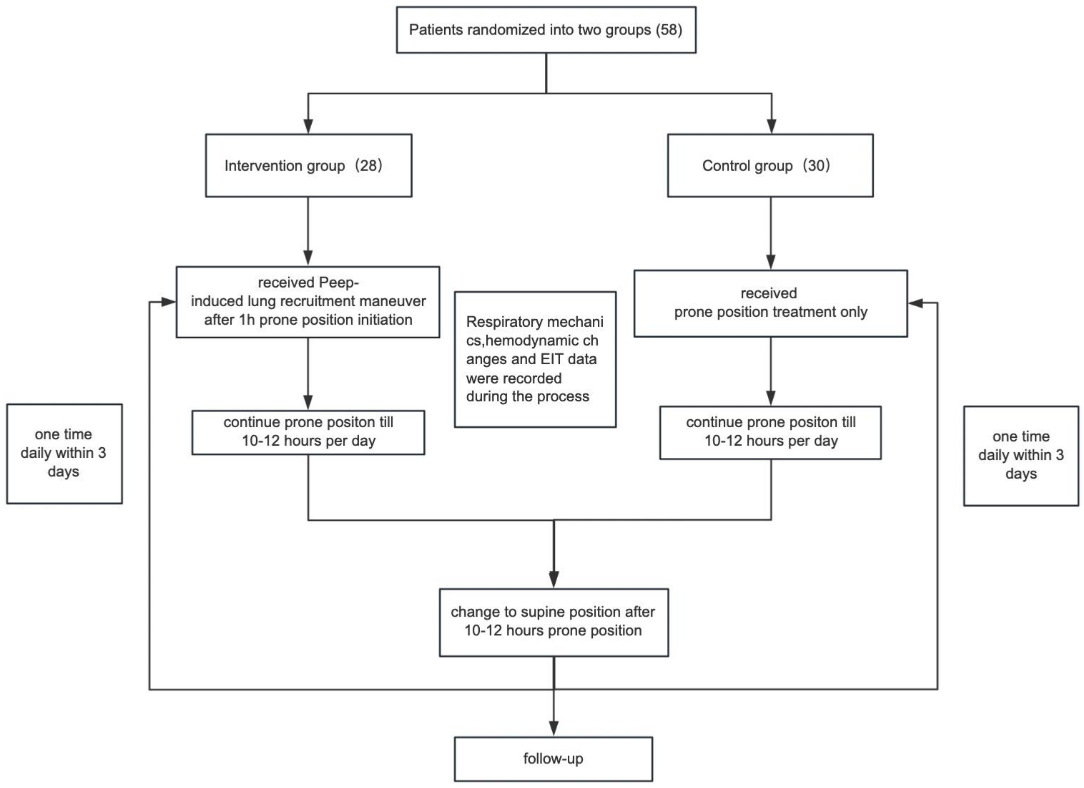

Supplementary figure S5. Electrical impedance tomography image. Blue regions reflect the tissue impedance of tidal breath, brighter colors mean higher ventilation in that area, and white colors indicate hyperinflation. The lung is divided into four regions named regions of interest (ROIs) in the horizonal layers; ROI 1 and ROI 2 are the ventral regions of the lungs, while ROI 3 and ROI 4 are the dorsal regions of the lungs. The ratio of the impedance in different ROIs to the global impedance is expressed as a percentage.

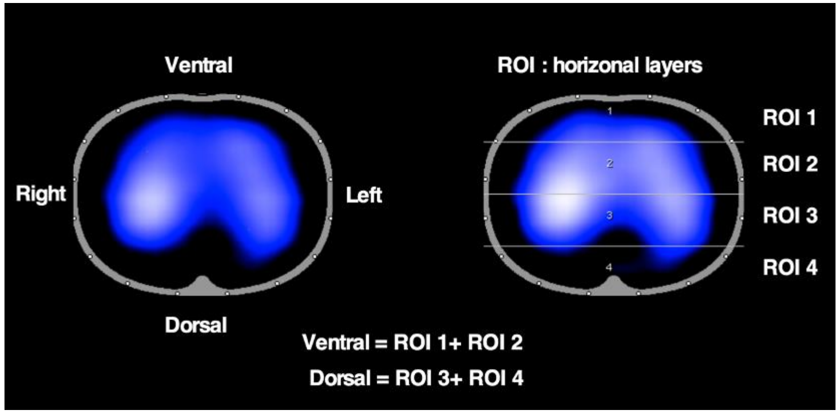

Supplementary figure S6. Regional distribution of lung ventilation in two groups. Differences in TV ROI 1, TV ROI 2, TV ROI 3, and TV ROI 4 between two groups in the horizonal layers. (A,B,C,D) Differences in ventral and dorsal regional distributions between two groups (E, F). T0: in the supine position; T1: 3 h after PP initiation/after LRM; T2: at the end of PP.

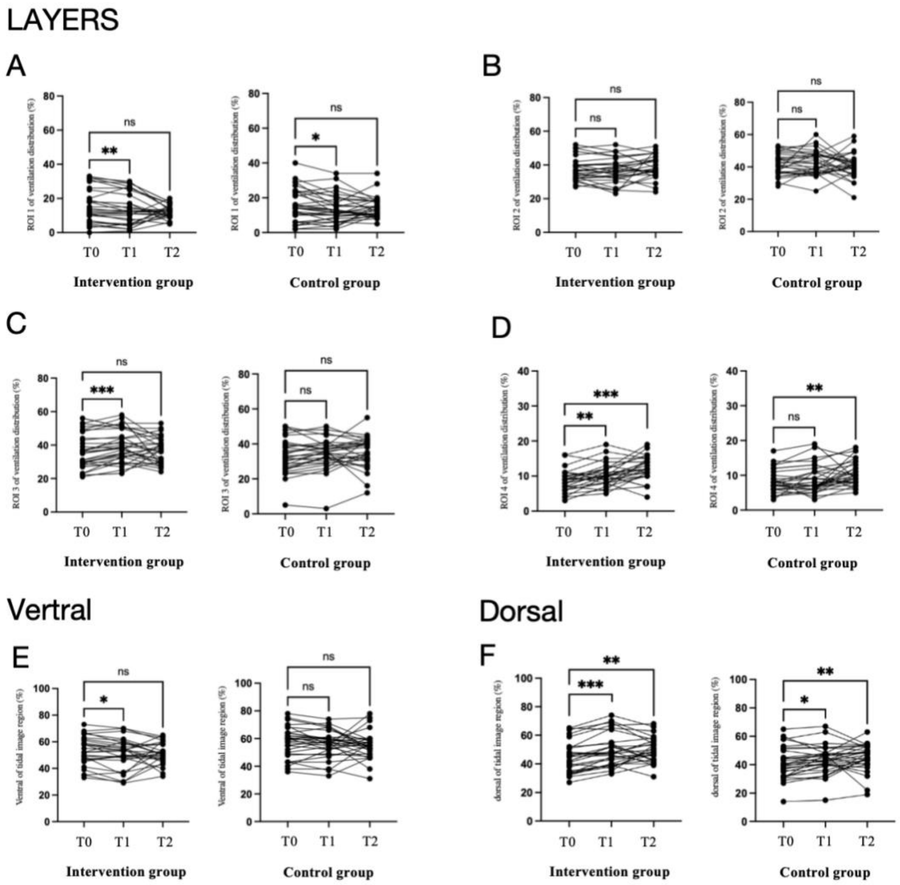

Supplementary figure S7. PaO_2 and PaO_2/FiO_2 at the three different time points between two groups; T0: in the supine position; T1: 3 h after PP initiation/after LRM; T2: at the end of PP. In the R/I >0.5 groups.

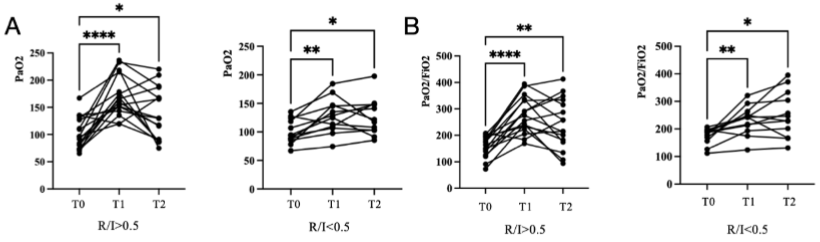

Supplementary figure S8. Regional distribution of lung ventilation in prone position combined with LRM in two subgroups, which is divided by the R/I ratio. With R/I=0.5 as the threshold, R/I >0.5 is defined as a high recruiter (A), and R/I <0.5 is defined as a low recruiter (B).

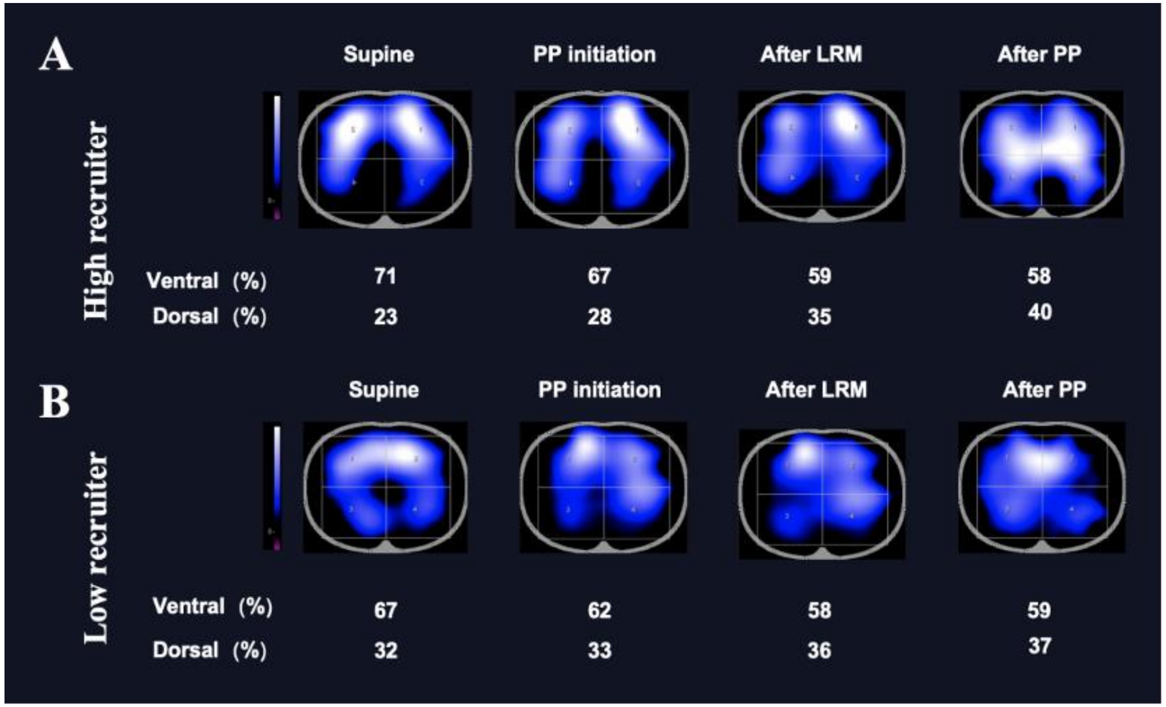

Supplementary figure S9. Comparison of the tidal ventilation distributions between two groups.

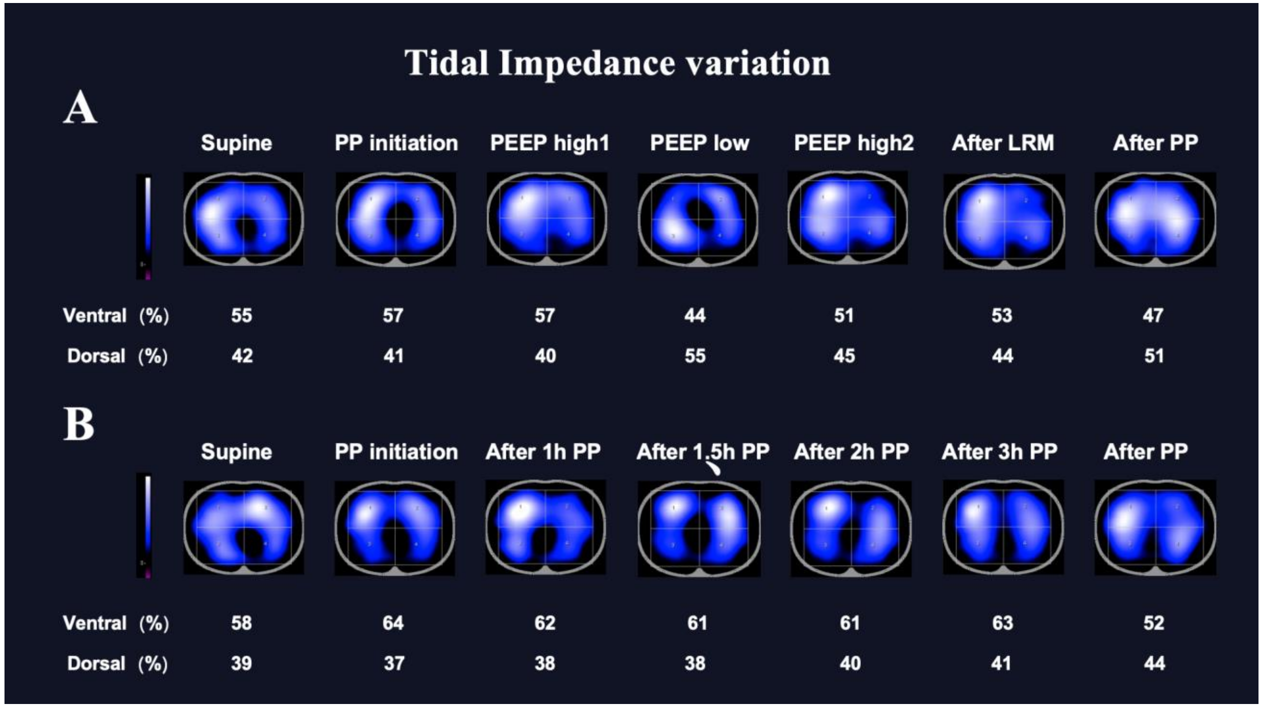

Comparing the tidal ventilation distribution of the intervention group (A) and control group (B) at seven time points during the first prone position evaluated by the EIT, in two representative patients. The ventral region refers to the sum of ROI 1 and ROI 2 (horizonal layers), while the dorsal region refers to the sum of ROI 3 and ROI 4 (horizonal layers).

Supplementary figure S10. Tidal impedance variation and compliance variation during the PEEP-induced lung recruitment.

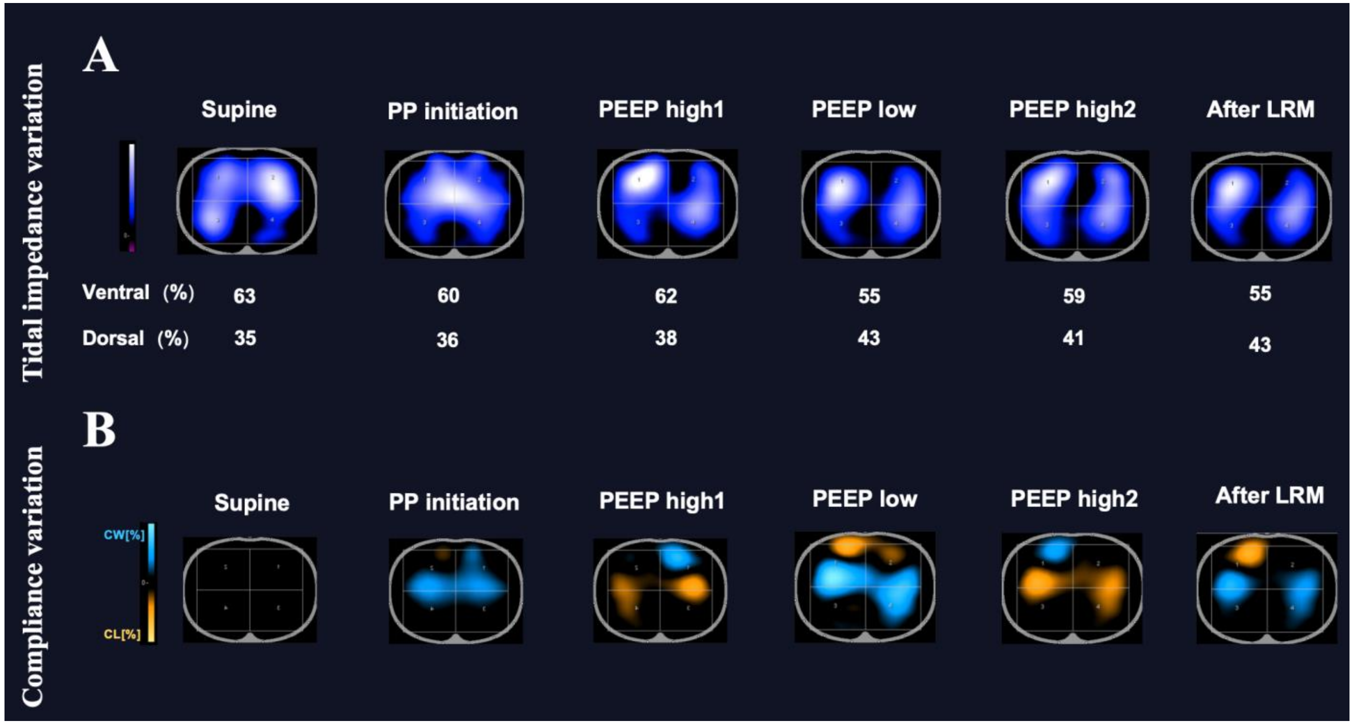

Tidal impedance variation (A) and compliance variation (B) during the PEEP-induced lung recruitment from a representative patient in the intervention group. In the compliance variation images, the green color indicates an increased compliance and orange means a decreased compliance.

Supplementary figure S11. Correlation between R/I ratio and A change in PaO_2/FiO_2 after LRM, B change in dorsal region distribution (%); after peep-induced lung recruitment, the higher the R/I ratio, the more the PaO2/FiO2 improved.

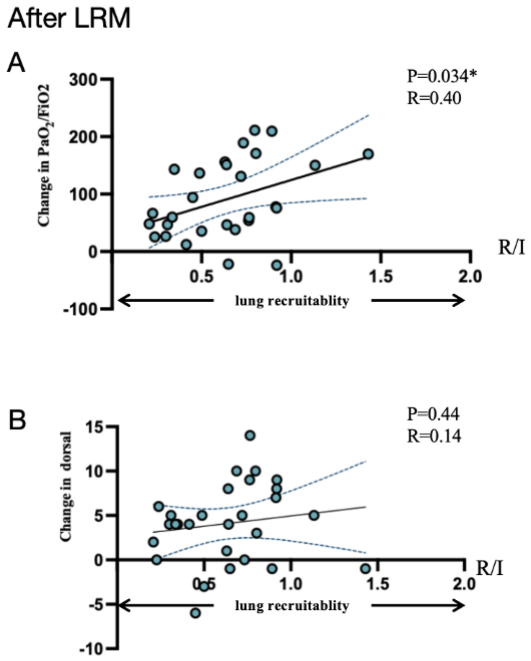

Supplement: Supplementary file 1 [file jcm-13-00853-s001.zip › jcm-2814929-supplementary.pdf]
